# Alveolar Bone Regeneration: Smart Biomaterials and Physical Stimulation

**DOI:** 10.1002/jbm.b.70082

**Published:** 2026-04

**Authors:** Allen Zennifer, Sai Sadhananth Srinivasan, Suranji Wijekoon, Sumit Yadav, Sama Abdulmalik, Sangamesh G. Kumbar

**Affiliations:** 1 Nebraska Translational Research Center (NTRC), Department of Growth and Development, College of Dentistry, University of Nebraska Medical Center, Omaha, Nebraska, USA; 2 Department of Advanced Specialty Sciences, James B. Edwards College of Dental Medicine, Medical University of South Carolina, Charleston, South Carolina, USA

**Keywords:** alveolar bone regeneration, bone graft substitute, cell regulatory pathways, osteogenesis, physical stimulation, smart stimuli-responsive materials (SSMs)

## Abstract

Large-area bone loss from disease, trauma, or congenital defects requires surgical procedures and bone grafting. Alveolar bone loss from severe periodontal disease and non-unions often demands immediate grafting. Treating large alveolar bone defects using grafts and substitutes is challenging due to the complex oral environment, infection risks, and unstable graft properties, which may compromise strength and bioactivity. Successful grafts must promote vasculature development and osteogenesis while maintaining mechanical stability at the graft site. Current bone graft substitutes are inadequate for optimal alveolar bone healing. New biomaterial technologies including additive manufacturing techniques enhance repair processes by developing anatomically equivalent implants that integrates better with host tissues, provide mechanical stability and activate innate healing mechanisms. Smart stimuli-responsive materials (SSMs), combined with exogenous physical stimulation, further advance this by triggering cell regulatory pathways, promoting bone mineralization, blood vessel development, and mechanical integrity. Electrical, magnetic, mechanical, ultrasound, and shockwave stimulations activate Ras, p38 kinase, PI3K/Akt, JNK, NF-κB, MAPK/ERK, Wnt, BMP, and VEGF pathways, enhancing osteogenic genes like Runx2, YAP, osteopontin, and osteocalcin to promote osteoinduction and osteogenesis. This article provides an in-depth literature analysis of smart biomaterials and stimuli-mediated alveolar bone repair and regeneration mechanisms. It also highlights the unmet needs of innovative biomaterials such as SSMs and explores strategies to manage the bone microenvironment, aiming to enhance clinical translation for large-area bone defects regeneration.

## 1 | Introduction

The alveolar bone is a specialized, dense bone structure in the jaw that provides support and anchorage for teeth. By forming the tooth sockets (alveoli), the alveolar bone plays a crucial role in withstanding the mechanical forces generated during chewing [1]. The alveolar bone undergoes continuous remodeling in response to functional pressures allowing it to adapt to changes such as tooth loss or orthodontic movement [2]. Its primary roles include supporting teeth, absorbing and distributing occlusal forces, and ensuring tooth stability, all of which are essential for oral health and overall jaw function [3]. However, the alveolar bone is susceptible to various diseases and injuries which can significantly impact both oral health and general well-being [4]. The main causes of alveolar bone loss are periodontitis, resorption, trauma, and injury [5–8]. Periodontitis is a severe gum disease that can lead to significant alveolar bone loss. It occurs when bacteria accumulate below the gum line, causing inflammation and infection. This inflammation damages the tissues and bones that support the teeth, potentially leading to tooth loss. Alveolar bone loss from severe periodontal disease and non-unions frequently requires immediate grafting, with nearly half of all adults in the U.S. needing some form of this treatment [9]. Particularly, periodontal disease affects nearly half of U.S. adults over 30, with rates rising with age [10]. Worldwide, severe periodontitis affects 10%–15% of adults and can cause major bone and tooth loss if untreated [11].

After tooth extraction, alveolar bone naturally shrinks, losing 25% of its width within the first year and up to 40%–60% of its height within three years [12]. This often complicates future dental procedures and may require bone grafts. Traumatic injuries, often from accidents or sports, are common among adolescents and young adults, affecting 25%–30% of school-age children and 15%–20% of adults [13, 14]. Such injuries often necessitate prompt intervention to prevent long-term complications. Osteoporosis, particularly in postmenopausal women, raises the risk of periodontitis and related bone loss by 86% [7]. Age-related bone loss also contributes to increased tooth loss in older adults [5, 15]. Additionally, among dental implant recipients, peri-implantitis affects up to 20%, leading to further bone loss if untreated [16]. These trends emphasize the importance of preventive care and early treatment to protect alveolar bone health and overall well-being. All these conditions that results in alveolar bone loss requires bone grafting and dental implants to help preserve the bone and improve the outcome of future dental treatments [17].

The alveolar bone is a part of the jaw that holds and supports the teeth. It anchors the roots of the teeth in sockets, giving them stability [8]. Structurally, it has four layers, where the periosteum is a connective tissue layer that covers the bone. The cortical bone, or compact bone, is the dense outer layer that provides strength. The cancellous bone, or trabecular bone, is a spongy inner layer that absorbs the pressure from chewing. The cribriform plate is a thin, dense layer that lines the tooth sockets and shows up as a clear line on X-rays [1]. The alveolar bone matrix is composed of collagen fibers, which provide flexibility, and minerals like calcium and phosphate, which contribute hardness and resistance to compression. Key cellular components of the alveolar bone include osteoblasts, which are responsible for bone formation; osteoclasts, which resorb bone; and osteocytes, mature bone cells embedded within the matrix that maintain bone health. Additionally, blood vessels and nerves traverse the alveolar bone, providing essential nutrients and signaling to the surrounding tissues [10]. These components enable the alveolar bone to remodel continuously in response to mechanical stresses from chewing and adapt to changes such as tooth loss or periodontal disease [1].

Bone is a dynamic tissue that undergoes continuous remodeling. Long bones are designed for structural support and movement, undergoing a slower rate of remodeling. In contrast, alveolar bone is specialized for dental support and is capable of rapid adaptation to local changes, such as tooth loss or orthodontic treatment. While both bone types are essential for overall health, their remodeling processes are tailored to meet their distinct functions in the body. Table 1 compares the structure and function of long and alveolar bone.

Both human bone and mandibular bone are designed to withstand specific mechanical stresses. However, the mandibular bone is particularly adapted to handle the repetitive forces of chewing and remodels rapidly to adapt to local changes [1, 10]. These properties make the mandible both durable and flexible, crucial for its unique functional demands. The mechanical properties of human bone, including the mandibular bone, vary based on factors like age, density, and location [18]. The cortical bone, which is denser, is stronger and stiffer than trabecular bone. Cortical bone typically has a compressive strength of 100–230 MPa and an elastic modulus of 15–25 GPa [19, 20]. Trabecular bone, on the other hand, has a lower compressive strength of 2–12 MPa [19, 20]. The mandibular bone, particularly in the alveolar region, is dense to support dental forces. Its cortical regions can withstand chewing forces with a compressive strength of 100–150 MPa [21]. However, its elastic modulus is lower at 10–15 GPa compared to long bones, allowing for some flexibility to handle varying chewing forces [22]. The mandible’s density varies across the jaw, with higher density in the alveolar region for dental support and lower density in less stressed areas [23]. These variations in density impact the mechanical properties across different regions of the jaw [24]. Therefore, it is necessary to develop bone graft substitutes or scaffolds that integrate structural, mechanical, and biological cues to support bone formation and remodeling. However, current gold-standard treatments, such as autografts, allografts, and alloplastic grafts, often fail to restore both structural integrity and biological activity, which remains a key challenge in modern regenerative dentistry.

Despite continuous evolution in innovative biomaterials and scaffold architectures, these solutions remain suboptimal and do not yet provide a complete restoration of bone function. This review provides a summary of the critical functional role and unique mechanical demands of alveolar bone, especially in the context of disease-or injury-related loss. By emphasizing the drawbacks of current bone grafting strategies, this review highlights the use of a new class of biomaterials—smart stimuli-responsive materials (SSMs) for the repair of alveolar bone defects. These materials become active and respond instantaneously to externally applied physical stimuli to regulate proliferative, anti-apoptotic, and immunomodulatory pathways specifically within the defect environment. Finally, this article discusses the current research status of SSM technologies, the obstacles they face, and feasible ways to translate them from the lab to the clinic using advanced technologies.

## 2 | Available Treatment Methods for Alveolar Bone Regeneration

**FIGURE 1 |:**
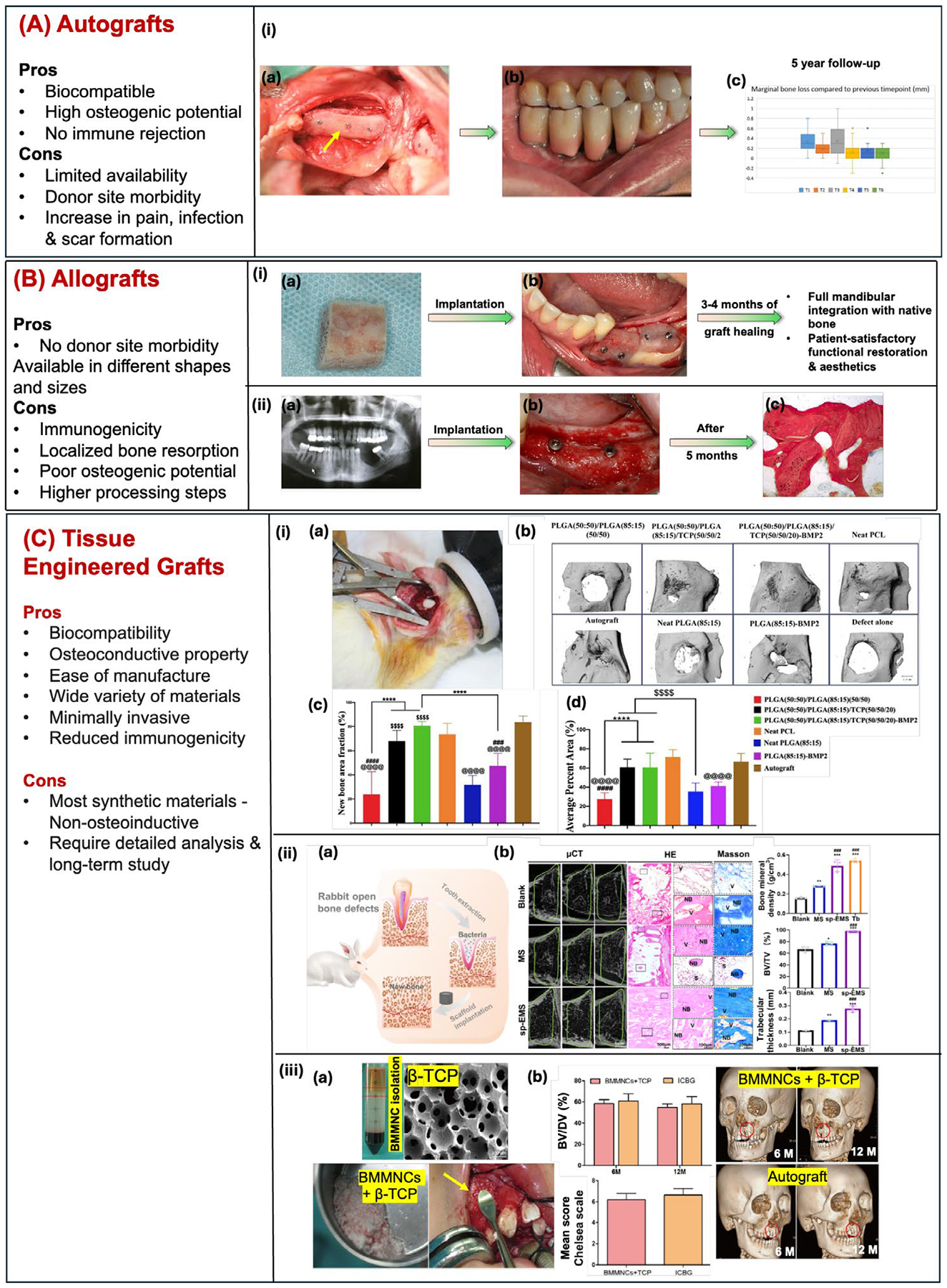
(A) (i-a) Autologous iliac bone and its particulates were fixed to the basal bone of right posterior mandible using titanium micro screws (indicated by yellow arrows). (i-b) Prosthetic fabrication and implantation was done within 3–5 months after the bone implantation surgery. (i-c) Post-surgical marginal bone loss (MBL) assessments for up to 5 years revealed reduced bone resorption compared to the previous time points. (B) (i-a) Allogeneic cortico-spongy bone blocks were implanted in alveolar bone defect, caused due to cyst or odontogenic inflammation and stabilized using Ti screws. (i-b) After 3 months, complete osteointegration and aesthetics were achieved without inflammation in all the patients; (ii-a) Posterior alveolar ridge defect developed due to trauma and infection. (ii-b) Grafting of mineralized allograft using screw tent-pole technique and positioned with 2 Ti screws. (ii-c) Histological analysis showed good bone formation after 5 months and restoration with functional load observed after 15 months of implantation. (C) Progression of tissue-engineered grafts from pre-clinical to clinical models: (i-a) Surgical implantation of PLGA(50:50)/PLGA(85:15) scaffolds with/without BMP-2 & TCP, PLGA(85:15) scaffolds with/without BMP2, neat PCL and autografts in a 5 mm critical sized rat mandibular defect model. (i-b) after 12 weeks, relatively higher bone volume fraction was observed in PLGA(50:50)/PLGA(85:15)/TCP (50/50/20) scaffold with BMP2 similar to neat PCL and autografts, confirmed from the μCT images. (i-c) New mature bone formation and infiltration was also seen within the defect area treated with PLGA (50:50)/PLGA (85:15) scaffolds incorporated with TCP & BMP2 than other scaffold groups. (i-d) Higher bone sialoprotein (BSP) expression observed for PLGA(50:50)/PLGA(85:15)/TCP with/without BMP2 scaffolds; (ii-a) Open mandibular 1st premolar bone defects were created in rabbits, followed by implantation of fibrillar collagen scaffold (mineralized scaffold, MS) and Ag nanowire-infused collagen scaffold (self-promoted electroactive minelarized scaffold, sp-EMS) and evaluated for new bone formation after 8 weeks. (ii-b) Micro-CT analysis revealed higher bone volume/tissue volume (~98.38%) with formation of native-like cortical & cancellous bone structures compared to EMS (~76.90%) and blank (~66.65%) groups. H&E and Masson trichrome staining also demonstrated higher blood vessel formation and collagen fibers similar to native architecture. Authors claim that these scaffolds produces mild currents through spontaneous electrochemical reactions to stimulate voltage-gated Ca2+ channels to enhance adenosine triphosphate (ATP)-induced actin remodeling and facilitate osteogenic differentiation of mesenchymal stem cells by activating the BMP2/SMAD5 pathway; (iii-a) Fabrication and implantation of tissue-engineered graft (bone marrow mononuclear cells (BMMNCs) mixed with β-tricalcium phosphate (β-TCP) (BMMNCs+β-TCP), indicated by yellow arrow) in unilateral cleft lip patients and compared with autograft (iliac crest bone graft, ICBG). (iii-b) Radiographic (indicated by red circle) and 8-point chelsea score analysis after 6 and 12 months for BMMNCs+β-TCP group showed complete repair in the defect site, comparable to autograft (Reproduced with permission from [26–31]).

Alveolar bone grafting (ABG) is essential for restoring bone volume and shape in the jaw following tooth loss, trauma, or congenital conditions like cleft palate, which can lead to significant bone defects [17, 25]. These defects impact dental function, aesthetics, and the success of procedures like dental implants. Conventionally, ABG involves reconstructing the bone defect by placing autografts, allografts, or engineered bone graft substitutes [17, 25] (Figure 1). Autografts, while effective, are limited in supply and require a secondary surgery [32]. Allografts are more abundant but carry risks of immune reactions and potential disease transmission [33]. Engineered graft substitutes, often derived from polymers, ceramics, or composites, avoid these limitations but may lack the natural bioactivity of biological grafts in promoting tissue regeneration [17, 25]. Efforts to enhance the bioactivity of engineered grafts include incorporating stem cells, bioactive factors, or external stimuli, either alone or in combination, to promote bone regeneration and osteointegration [34, 35]. These approaches help maintain bone height, provide structural support, and create a stable foundation for future dental restorations, ultimately supporting oral health and functionality [17, 25].

### 2.1 | Autografts

Autogenous bone grafts (autografts) remain the clinical gold standard treatment for mandibular reconstruction and are categorized as non-vascularized & vascularized bone grafts. Vascularized bone grafts are highly effective for promoting large-volume bone regeneration and achieving excellent functional and aesthetic results. With a survival rate exceeding 90%, these grafts significantly reduce graft resorption and promote revascularization, leading to superior healing outcomes [36]. The non-vascularized autografts are used for small and medium-sized bone defect reconstructions. These grafts harvested from areas of less vasculature, are implanted in vascularized bone defects. However, their success depends upon bone defect size, extent of its vasculature and bone stability [37]. Often bone autografts are harvested from iliac crest of the pelvic bone, ribs, tibia, fibula flap in the lower limbs, scapula in the shoulder region, calvarium, and radius in the forearm. Essentially all autografts enhance the success rate of bone grafting due to their bone remodeling properties including osteoconductive, osteoinductive and osteointegrative nature [38]. However, the need for a secondary surgery and the limited availability of autografts make their use challenging in complex and large-area bone reconstructions [33, 39]. A recent clinical study involving two patients on mandibular reconstruction utilized fibula grafts after tumor removal. One had ossifying fibroma, and the other had ameloblastoma. The fibula grafts were taken from their left legs. Post-surgical micro-CT scans showed good integration of the grafts into the mandibular defects. Both patients reported satisfactory functions, including chewing, swallowing, mouth opening, and speech, along with improved aesthetics [40].

### 2.2 | Allogeneic Bone Grafts

Allogeneic bone grafts harvested from donors or processed bones for human corpses are alternatives for large area and total mandibular reconstruction where the use of autografts is not viable. Before bone graft harvesting and implantation, a thorough preoperative assessment is essential to minimize post-surgical complications. This assessment typically includes a physical examination, imaging studies (X-rays, ultrasound, CT, and MRI), and laboratory tests to evaluate microbial and serological factors [27]. Demineralized bone matrix (DBM) and allogenic bone grafts are used for mandibular reconstruction surgeries. DBM is a putty obtained by removing hydroxyapatite (~92%–99%) and it can be easily adapted into complex shapes in non-load bearing areas. DBM is essentially collagen, sodium hyaluronate and other proteins and growth factors to promote osteogenesis. A patient who underwent complete mandibulectomy for Langerhans cell tumor received a successful implant of a lyophilized mineralized mandible from a deceased donor. Two years post-surgery, the patient demonstrated satisfactory facial contour restoration and minimal bone resorption in the mandible, as confirmed by radiological examinations [41]. These bone grafts are immediately radiation-sterilized, freeze-dried and can be obtained in the form of powder, chips, blocks, paste, blades, putty and injectables, exhibiting osteoconductive and osteoinductive properties [42]. In a study, these demineralized bone particles were mixed with gelatin to form a bone paste. In vitro seeding of human bone marrow-mesenchymal stromal cells (BMSCs) enabled attachment and osteogenic differentiation. These matrices supported the bridging of the critical-size bone defect in rat calvaria further confirm its potential toward oral and maxillofacial bone grafting [43]. However, some reports highlight the clinical limitations of bone allografts, including potential biocompatibility issues, risk of disease transmission (e.g., hepatitis and HIV), immune rejection, reduced osteogenic potential due to higher processing steps, and poor or delayed integration with host tissues compared to autografts [44].

### 2.3 | Tissue-Engineered (TE) Bone Grafts

Engineered bone graft substitutes are fabricated from natural polymers such as collagen, chitosan, gelatin, silk fibroin and polyhydroxyalkanoates (PHA) to name a few. Synthetic polyesters such as polylactic acid (PLA), polyglycolic acid (PGA), poly(L-lactide-co-glycolide) (PLGA) are very popular due to their FDA approval status in biomedical devices. These polyester-based grafts possess bone-equivalent mechanical properties, ideal for load bearing applications. However, their degradation process in physiological environment result in acidic byproducts that greatly hinders tissue regeneration ability. Additionally, the degradation rates of these copolymer-based scaffolds varies depending on their ratio and molecular weight [31], which may not be suited for load bearing applications. To avoid this challenge and to mimic the natural bone structure, various inorganic bioceramics like hydroxyapatite (HA), tricalcium phosphate (TCP), biphasic calcium phosphate (BCP), and bioactive glass (e.g., SiO_2_, Na_2_O, CaO, and P_2_O_5_) have been utilized as engineered bone graft substitutes [45]. Some of these components like TCP neutralize the acidic degradation byproducts of PCL, PLA, PGA and PLGA and enhance the mechanical properties [31].

A few load-bearing applications utilize metal derived grafts and are often based on stainless steel Co, Ti, and Ti-Ni alloys. Bone is a complex composite material composed of organic collagen and inorganic hydroxyapatite. To replicate these properties, many bone graft substitutes are designed as composite materials. Porous composite grafts and scaffolds can be fabricated using various techniques, including gas foaming, emulsion freeze-drying, electrospinning, thermally-induced phase separation (TIPS), solvent casting and particulate leaching, microparticle sintering, and 3D printing [46]. These grafts can be prepared using wide range of materials tailored to meet specific requirements based on the bone resorption rate and healing conditions. They also offer unlimited availability and lower risk of infection, disease and immune reactions compared to biological grafts. Some of the FDA approved dental bone graft substitutes used for reconstruction include Creos xenoprotect, BioGuide, Smart Bone, Augment (bone graft with PDGF), Infuse (bone graft with BMP-2) and Osigraft (bone graft with BMP-7) [47]. Engineered bone graft substitutes, despite their advantages over biological grafts, still face challenges. These include suboptimal mechanical properties, undesirable resorption rates, toxic degradation products, and poor vascularization, all of which can hinder tissue ingrowth and integration with host bone. These limitations can compromise the long-term success of dental restorations and functional outcomes [48]. The unregulated release of growth factors from FDA-approved bone graft substitutes often leads to the release of supramolecular doses, resulting in inflammatory complications and suboptimal healing outcomes. These suboptimal release profiles can hinder the desired therapeutic effects and potentially lead to adverse side effects [49]. Using traditional and engineered bone graft substitutes for mandibular reconstructions presents several challenges, including difficulties with proper graft positioning, inadequate vascularization, limitations in manufacturing complex structures like condyles, and the need for extensive surgical incisions [50].

In addition to growth factors or drug molecules, stem cells are also essential for stimulating and secreting the expression of osteogenic and vascular markers (e.g., Runx2, ALP, osteocalcin, BMPs, VEGF), regulating inflammatory microenvironment, and capable of differentiating into osteoblasts, chondrocytes, adipocytes, and fibroblasts [51, 52]. Multipotent stem cells including those from the periodontal ligament (PDLSCs), alveolar bone (AB-MSCs), and bone marrow (BM-MSCs)—respond to external stimuli to improve proliferation, promote osteogenic differentiation, and regulate the remodeling phase. These stimuli include mechanical forces like cyclic strain and shear stress, as well as electrical, ultrasound, and electromagnetic fields [53]. For example, a study focused on how low-intensity pulsed ultrasound (LIPUS) can enhance the homing of BMSCs to the site of injury and its potential to accelerate alveolar bone repair [54]. Isolated BMSCs from rats labeled with luciferase and green fluorescent protein (GFP) by lentivirus in vitro where 1 × 10^6^ Luc-GFP labeled BMSCs were injected into rats with a periodontal bone defect on the mesial area of the maxillary first molar. LIPUS was delivered directly via ultrasonic probe on the periodontal defect site using the following parameters: frequency—1.5 MHz, pulse duration—200-μs, pulse repetition frequency—1.0 kHz and intensity—30 mW/cm^2^. The rats were sacrificed eight weeks after surgery to perform micro-CT and immunohistochemistry staining to evaluate alveolar bone regeneration. The study found that BMSCs differentiated into osteoblasts and were successfully transfected with a GFP marker. Bioluminescence imaging revealed that most BMSCs were trapped in the lungs, with fewer cells homing to the alveolar bone defect. However, the BMSCs/LIPUS group showed significantly higher BMSC homing and new bone formation than the control (non-LIPUS treated) group. These findings suggest that LIPUS can enhance the therapeutic potential of BMSCs for periodontal bone repair. In another study, the impact of duty cycles and the proportion of time during an on/off pulse period is determined when ultrasound is generated under ultrasound stimulation [55]. This study employed LIPUS at 1 MHz with 20% and 50% duty cycles to treat human alveolar bone-derived mesenchymal stem cells (hAB-MSCs). The treatment intensity was set at 50 mW/cm^2^, and the cells were exposed for 10 min per day. The findings demonstrated that the cell survival of hAB-MSCs exposed to duty cycles of 20% and 50% was comparable to that of the control. Following LIPUS therapy, increased mineralized nodule formation and upregulation of osteogenic genes (Col-I and ALP) were observed. hAB-MSCs exposed to LIPUS exhibited enhanced osteogenic differentiation. This suggests that LIPUS may influence cell adhesion molecules and promote osteogenesis. Previous studies have confirmed the efficacy of LIPUS in accelerating bone healing in cases of distraction osteogenesis, delayed unions and nonunion, both in animal models and human patients [56–61]. These studies were conducted without the use of tissue-engineered acellular/cellular scaffolds. The clinical application of both allogeneic and autologous mesenchymal stem cells (MSCs) has been limited due to concerns about potential tumorigenicity and metastasis with long-term efficacy, insufficient supporting evidences, and ethical as well as regulatory challenges [62, 63]. Further, detailed studies are crucial to optimizing the application of ultrasonic stimulators, as challenges persist regarding potential adverse effects linked to specific intensity, duration, and duty cycle settings.

**FIGURE 2 |:**
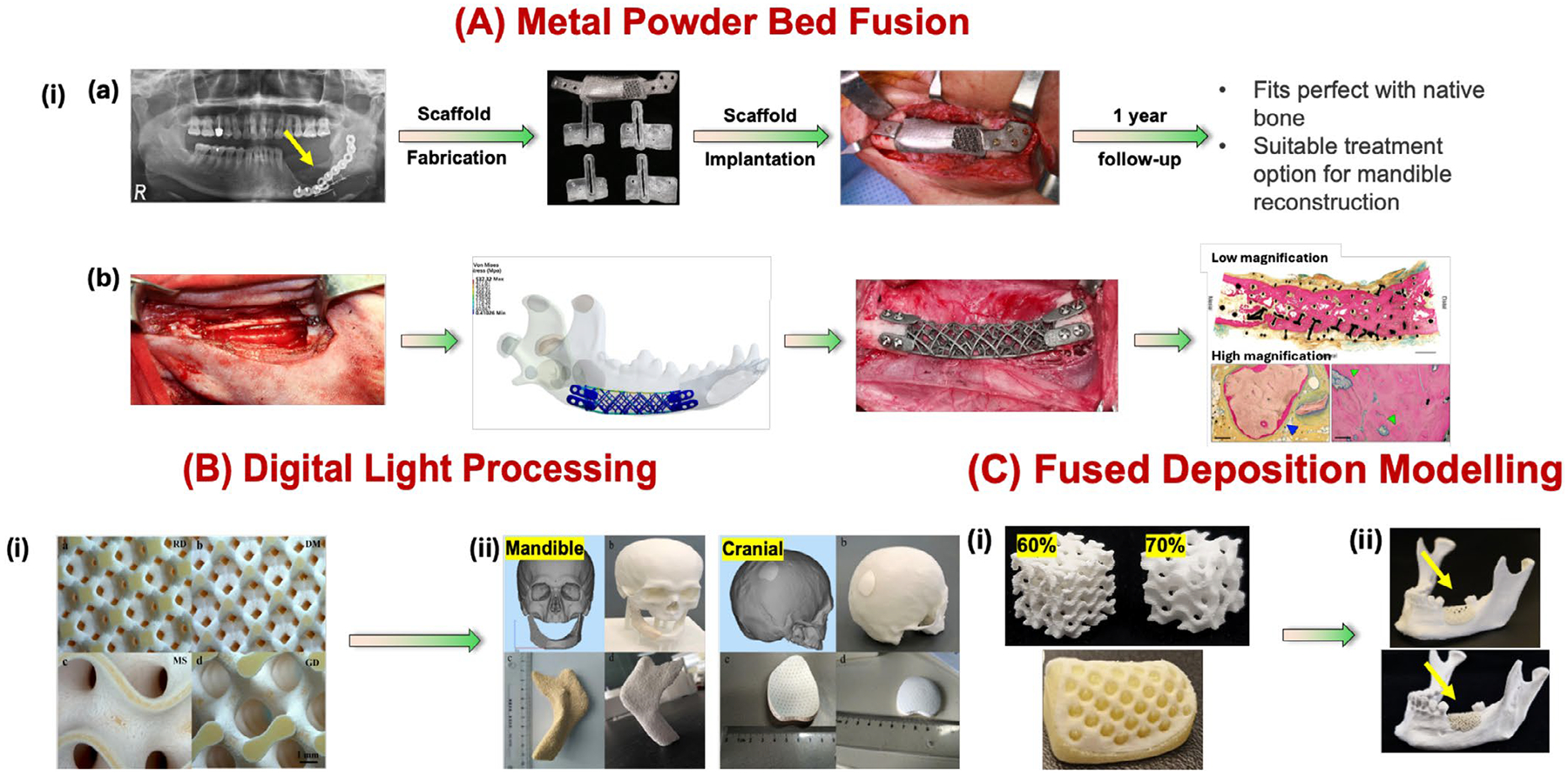
(A) (i-a) Customized 3D-printed resection guide and titanium mandibular implant was fabricated to implant in a severe fracture in the left mandible (indicated by yellow arrow) with osteoradionecrosis (ORN) and fixed with biocortical screws. After 1 year follow-up post-surgery, no evidences of postoperative infection or foreign body reaction were observed (Reproduced with permission from [64]). (i-b) A 4 cm critical-sized segment mandible defect in beagle dogs were created (indicated by yellow arrow), followed by 3D modeling of Ti-mesh implant as per the bone anatomy and printed using SLM. The 3D-printed Ti-implant was fixed along with the autogenous crushed bone mixture in the defect site. Methylene blue/acid fuchsin staining after 18 months demonstrated calcified new bone formation (stained in bright pink) with higher bone/tissue volume (distal-30.22, middle-22.758, and mesial-18.93) along with native bone integration (low magnification: stained in brown, high magnification: blue triangles) and Ti-mesh scaffold (stained in black), and the bone substitute was stained brown in the slides and Havers’ systems formation (high magnification: green triangles). (B) (i) Optical images of the sintered β-TCP scaffold having four different pore configurations, prepared using DLP technique; (ii) Personalized, biomimetic and sintered β-TCP ceramic bone 3D-construct for porous trabecular bone and crania defect was developed after 3D modeling (C) (i) Additively manufactured bioresorbable composite scaffolds were printed with different shapes and porosities; (ii) Visual assessment of patient-specific 3D-printed graft (indicated by yellow arrows) printed with different infill patterns for alveolar bone defect were fixed on a 3D-printed mandible model (Reproduced with permission from [65–67]).

Rapid prototyping (RP) or additive manufacturing (AM), another technological advancement in digital manufacturing, has shown the potential to overcome many challenges associated with traditional bone graft substitutes and scaffolds fabrications. By utilizing 3D imaging techniques like CT and MRI, the defect site can be accurately visualized and reconstructed into a 3D virtual model. This model can then be further refined using computer-aided design (CAD) and computer-aided modeling (CAM) software to create a patient-specific design that matches the exact dimensions and symmetry of the original anatomy. Finally, a customized graft can be manufactured using biocompatible materials and 3D printing techniques. Figure 2 presents the available 3D printing techniques to create anatomically equivalent 3D-printed alveolar bone grafts for the surgical procedures. Until now, 3D printing techniques like metal powder bed fusion (including SLS, EBM, and SLM), fused deposition modeling (FDM), and digital light processing (DLP) have been employed to fabricate both dental and orthopedic implants [68]. Table 2 shows the list of advantages and disadvantages of each available AM technique, which describes the mandibular bone tissue regeneration.

Following successful implantation of bone grafts, the most important clinical challenge is the infection in the defect sites caused due to the several strains of *Staphylococcus aureus*, *Streptococcus mutans*, *Staphylococcus epidermidis*, *Escherichia coli*, and *Candida albicans* [81, 82]. Thus, these AM techniques can be combined with clinically proven anti-infective agents or strategies to develop customized bone grafts that not only prevent infection but also support osteogenesis through bioactive scaffolds [83]. Anti-microbial drugs (e.g., vancomycin, ciprofloxacin, gentamycin [84, 85]), oligopeptides (e.g., LL37 [86]) and nanoparticles (e.g., Ag, Au, Cu [87–89]) were surface-coated or encapsulated within biomaterial inks of the 3D-printed implants. Though these scaffolds undergo several processing steps, they retain their antimicrobial properties and show continuous release of antimicrobial agents to avoid biofilm formation by inhibiting bacterial adhesion at early stages. For instance, a piezoelectric filler material—BTO was incorporated in photocurable gelatin methacryloyl (GelMA) hydrogels to evaluate antibacterial and bone tissue regeneration property. Results showed significant inhibition of *P. gingivalis* growth and biofilm formation. The BTO-loaded hydrogel generates electric charges under cyclic mechanical loading (repeated stretching and compression), which induce electrostatic damage to bacterial membranes and promote the formation of reactive oxygen species (ROS), ultimately resulting in bacterial cell death. Importantly, these materials exhibited minimal toxicity, bone mineralization and osteogenic gene expression in periodontal cells, highlighting their suitability for biomedical applications [90]. Zhang et al. developed a grid-patterned circular (6 mm diameter and 1 mm thick) scaffold using β-tricalcium phosphate/poly (lactic-co-glycolic acid) composite ink infused with an antibacterial drug component (chlorhexidine (CHX) encapsulated graphene oxide (GO) nanosheets) and a functional osteogenic oligopeptide (p24) via cryo-3D printing and dip coating methods. The 3D printed scaffolds displayed the following properties: Porosity: 75%–85%, higher compressive stress: ~15 MPa, highly hydrophilic: ~50 degrees and CHX release: an initial burst release (69.33% ± 4.69%) within 48 h followed by sustained release for up to 14 days. These physicochemical properties resembled the trabecular structure in the mandibular bone. Based on the in vitro results, these scaffolds also provided a conducive environment for bacterial growth inhibition and osteoblast infiltration. After implanting the CHX@GO-treated scaffolds in a 6 mm rat mandible defect infected with *S. aureus* for a week, bacterial colony forming units (CFUs) were significantly lowered compared to non-treated and non-drug loaded scaffold groups, resulting in reduced inflammation. Both p24 peptide and phosphate ions released from TCP nanoparticles induced the differentiation potential of bone marrow derived stem cells into osteoblasts as they showed increased mineralization parameters—bone mineral density, bone volume/tissue volume and higher expression of osteogenic markers—ALP, collagen I within 8 weeks. Hence, this research may provide an effective treatment strategy to address bone defects with microbial infection [91]. Overall, these systems were engineered via controlled nanoparticle synthesis to provide synergistic, sustained antibacterial activity, including effectiveness against drug-resistant strains, while maintaining biocompatibility for regenerative applications.

Digital technologies also enable virtual surgical planning, allowing clinicians to visualize the procedure and develop a detailed surgical plan. This virtual planning can significantly reduce actual surgical time and costs. The accuracy and efficacy of the 3D-printed customized titanium meshes were investigated in 20 patients with bone defects both minor (defect surface area: ≤ 150 mm^2^; 1–2 adjacent front teeth/premolars missing) and major (defect surface area: > 150 mm^2^; adjacent ≥ 3 teeth or ≥ 2 M missing) [92]. Before surgery, CBCT imaging was used to create 3D models of the patient’s anatomy. These models were then analyzed using 3D CAD software to plan the optimal placement of implants and dental crowns. This digital planning process was crucial for guiding the bone augmentation procedure. Based on the virtual 3D bone model, patient-specific titanium meshes were 3D-printed using medical-grade titanium alloy powder and laser-based additive manufacturing techniques. Additionally, a photosensitive resin-based guide plate was 3D-printed using digital light processing. Following surgical implantation of the 3D-printed bone graft into the alveolar bone defect, the patient was monitored for up to 9 months. During this period, the soft tissues healed completely without any signs of infection. Additionally, precise alignment between the post-bone graft and the original CAD models was ensured to accurately assess the outcomes for both minor and major defects [92]. However, significant loss of anatomical structures can lead to slight discrepancies between the virtual and actual bone volumes and contours during bone augmentation. Further research is needed to assess the impact of defect size and complexity on the accuracy of 3D-printed bone grafts and their clinical outcomes [93]. Other significant barriers to use AM technology in clinical settings include limited biomaterial ink/bioink choices, require specialized software to develop intricate 3D bone structures at higher resolution, difficulty in achieving sufficient vascularization in large or dense 3D-printed bone grafts, additional post-processing steps and lack of standardized regulatory and quality control measures [94].

Despite advances in current treatment strategies, including autografts, allografts, xenografts, and engineered bone substitutes, alveolar bone regeneration outcomes remain suboptimal, particularly in large or compromised defects. While traditional grafts continue to be widely used, they are frequently limited by donor site morbidity, immune response, infection risk, inadequate vascularization, and inconsistent long-term integration [33, 39, 44]. Tissue engineering approaches that incorporate biomaterials, cells, and growth factor alternatives were developed to overcome these limitations; however, they have largely produced incremental improvements rather than consistently robust regeneration [47]. A key barrier to effective alveolar bone healing is the persistently inflammatory and hostile oral microenvironment, characterized by microbial burden, mechanical instability, and repeated tissue insult. Bioactive molecules such as BMPs and other growth factors often exhibit short half-lives, rapid diffusion, and loss of bioactivity, limiting their therapeutic effectiveness and, in some cases, increasing adverse effects [49]. These challenges are further exacerbated by underlying systemic conditions such as diabetes, aging, and chronic inflammation, which substantially impair regenerative capacity and reduce the success of conventional graft-based therapies. In parallel, physical therapy modalities, including electrical stimulation (ES), ultrasound, and magnetic stimulation, have demonstrated the ability to promote tissue regeneration by modulating immune responses, enhancing neovascularization, and stimulating extracellular matrix synthesis and remodeling [95–99]. Importantly, these modalities act by inducing cells to secrete endogenous cytokines and growth factors, thereby promoting healing without reliance on supraphysiological doses of exogenous biologics. However, a major limitation of these approaches is the difficulty of delivering uniform, sustained, and localized stimulation, particularly in complex anatomical sites such as the oral cavity, where large or irregular defects can lead to inconsistent therapeutic outcomes [100, 101]. These limitations underscore the need for smart stimuli-responsive materials (SSMs) that integrate biomaterial design with externally applied stimulation. SSMs provide a means to distribute physical stimuli throughout the defect site, regulate local cellular responses, and achieve more predictable regeneration. Furthermore, combining short-acting bioactive molecules to initiate healing with sustained physical stimulation to guide immune modulation, angiogenesis, and matrix remodeling offers a synergistic strategy to enhance alveolar bone regeneration [100, 101]. Together, these considerations provide the conceptual foundation for transitioning from conventional graft-based approaches to SSM-enabled regenerative strategies, which are discussed in the following sections.

## 3 | Influence of Smart Materials on Cell Regulatory Pathways

The application of scaffolds, growth factors, cells, and other substances in bone regeneration is a well-known strategy and widely used in tissue engineering. Scaffolds provide a three-dimensional structure that supports cell attachment, proliferation, and differentiation. They are designed to mimic the natural extracellular matrix, creating an environment conducive to bone growth [102]. Generally, metals, natural & synthetic polymers, ceramics, and composites have been used to fabricate bone scaffolds/grafts for engineering damaged bone tissues. Some of the FDA-approved graft materials include ceramics (e.g., calcium phosphate, calcium sulphate, tricalcium phosphate, biphasic calcium phosphate), inorganic materials (e.g., hydroxyapatite), metals (e.g., titanium and its alloys), natural polymers (e.g., collagen), and synthetic polymers (e.g., polymethylmethacrylate (PMMA)). Growth factors, such as bone morphogenetic proteins (BMPs), platelet-derived growth factor (PDGF), and transforming growth factor-beta (TGF-β), play a crucial role in bone healing by stimulating cell activity and promoting new bone formation [103–106]. Among these, BMP-2 is the FDA-approved growth factor for bone regeneration, including dental and alveolar bone applications [107]. BMPs play a key role in promoting osteoblastic differentiation. The action of BMPs likely involves the transcription factor Osterix, which functions downstream of Runx2 [108]. The delivery of exogenous growth factors has several drawbacks, including high dose requirements, short half-life, protein instability, high costs, and potential side effects [109]. To address this challenge, they are often combined with bone grafts like collagen sponges. These sponges act as a scaffold for the growth factor, delivering it to the bone defect site and can improve healing outcomes and restore alveolar bone structure and function after tooth loss or injury [103–107]. These growth factors can help recruit cells, stimulate blood vessel growth, and enhance the integration of bone grafts or scaffolds. Current research aims to reduce the concentration of growth factors in bone graft substitutes to achieve an optimal dose that promotes healing while preserving bioactivity [109]. While BMP-2 is well-established, other growth factors, such as PDGF and TGF-β, are still under investigation in preclinical [103–106]. Overall, these bone graft materials have revolutionized the medical industry for the past few decades, possessing advantages such as adequate porosity, good biocompatibility, and bone-mimic mechanical strength. However, these types of implants also have potential limitations such as high brittleness & rapid resorption rate (for ceramic-based materials), higher stiffness, infections & toxicity (for metal particles), and hydrophobicity with poor wettability and mechanical strength (for synthetic polymers), that might restrict their long-term usage. Thus, these issues can be overcome by tailoring these materials on demand depending upon the microenvironment of the damaged bone. In this regard, a new class of materials has been developed to detect and react immediately to external or internal stimuli in a spatiotemporal manner, referred to as smart stimuli-responsive materials (SSMs). These materials change their physical or chemical properties in response to specific pathological conditions or applied physical stimulation to induce a desired biological effect on certain type of cells or tissues. These SSMs have recently been explored in various biomedical fields, such as drug delivery, tissue regeneration, biosensors, bioimaging modalities, etc. The idea of using smart stimuli-responsive biomaterial scaffolds in bone tissue engineering is primarily to modulate the bone microenvironment on-demand and induce bone regeneration and remodeling with adequate vascularization.

**FIGURE 3 |:**
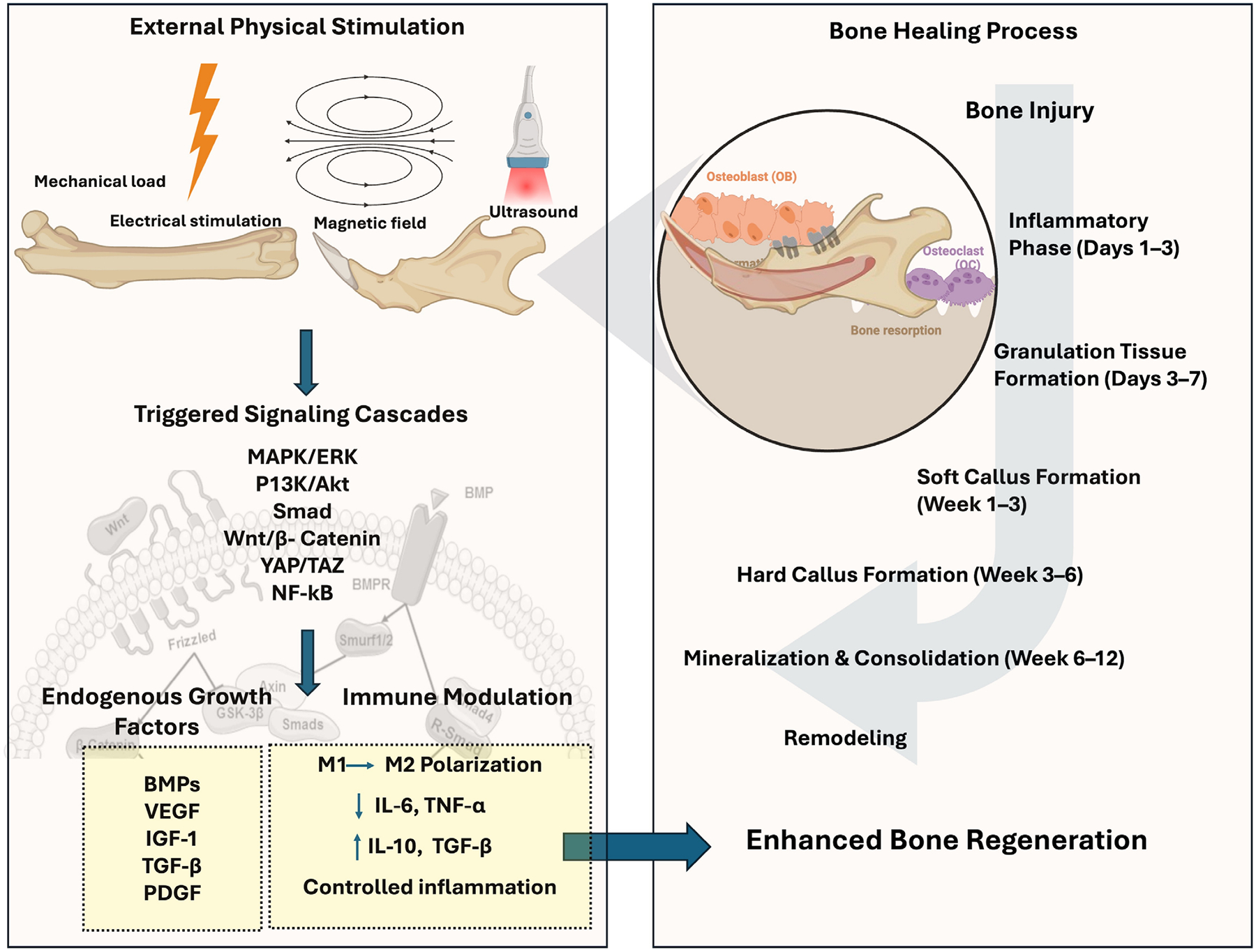
External physical stimulation strategies and their role in bone regeneration. Mechanical loading, magnetic fields, and ultrasound activate mechanotransduction pathways in bone cells, triggering signaling cascades such as Wnt/β-catenin, Smad, and MAPK. These pathways enhance endogenous secretion of growth factors (e.g., BMPs, VEGF, IGF-1) and cytokines, driving osteoblast differentiation, osteocyte activity, and mineralization while modulating the immune microenvironment to balance osteoclast and osteoblast functions. Collectively, these stimuli promote coordinated bone remodeling and regeneration. Created with BioRender.com.

Physical therapy modalities, such as mechanical loading [96], electrical stimulation (ES) [97], magnetic fields [98], and LIPUS [99], have been progressively used in clinical practice [95] and have shown potential in promoting bone tissue healing and regeneration (Figure 3). These external stimulations have demonstrated significant benefits for both soft and hard tissue repair. While physical stimulation methods are promising for non-invasive bone regeneration therapies, precise delivery at optimized doses remains challenging, leading to variability in healing outcomes [100, 101]. Hence, smart materials are engineered to respond to external stimuli either reversible or irreversible and efficiently distribute energy to defect sites.

**FIGURE 4 |:**
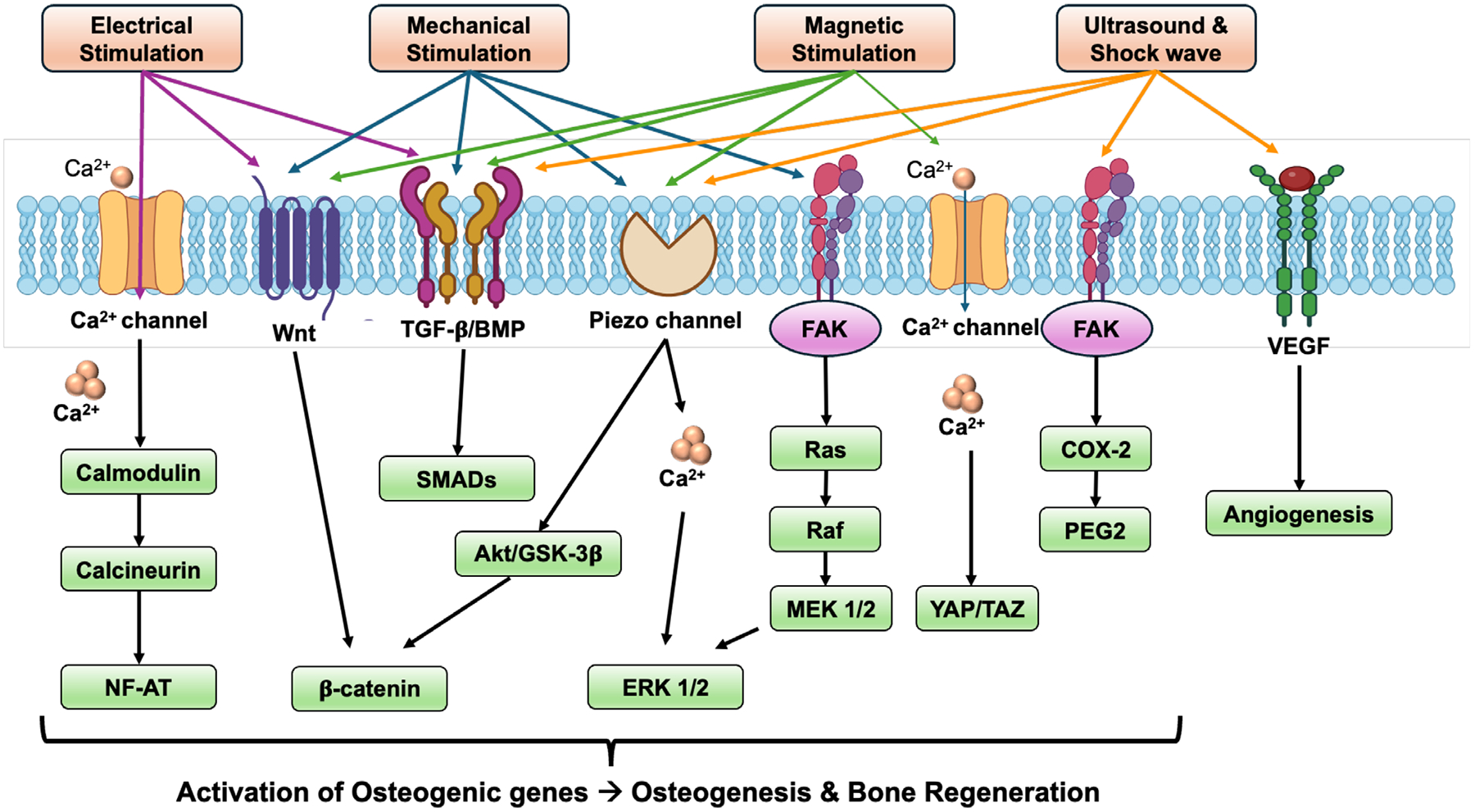
Schematic representation of cellular response and activation of signaling pathways by externally stimuli to activate osteogenic-specific pathways and promote bone differentiation and regeneration. Created with BioRender.com.

A thorough knowledge of physiological and pathological characteristics in the damaged or infected bone is important to develop novel, multi-faceted SSMs. In addition, the applied external cues should not damage the developed SSMs and cause toxicity to the tissues. Mostly, these SSMs are prepared as nanocarriers and incorporated in the scaffold matrices. Upon application of physical stimulation such as electric field, magnetic field, ultrasound, and mechanical forces on the SSM-based scaffolds, these materials alter their structure to directly induce the bone cells/tissues to cause physical, chemical, and biological effects by activating several cell-signaling pathways to facilitate bone regeneration with adequate angiogenesis and innervation (Figure 4). Integrating external stimulations with smart materials can enhance the natural healing process and improve the effectiveness of grafting materials, making them a valuable addition to regenerative medicine [100, 101]. Further, in the process of bone healing, angiogenesis also plays a crucial role by supplying essential nutrients for osteogenesis and facilitating bone tissue regeneration [110]. Therefore, unidirectional interactions between immune cells and bone cells play a crucial role in bone remodeling in order to facilitate bone healing [111]. Immune cells, such as macrophages, influence the osteogenic activity of bone cells by secreting cytokines and growth factors that either promote or inhibit bone formation, depending on the phase of healing. These complex cellular interactions ensure proper healing, remodeling, and the restoration of bone integrity [110, 111]. Some of the available SSMs used for alveolar bone regeneration were explained in the subsequent sections.

To provide a clear and accessible comparison of the smart stimuli-responsive materials (SSMs) discussed in this review, we have compiled a summary table (Table 3). This table organizes the SSMs by material type, stimulation modality, target application site, observed biological outcomes, and translational status. It complements the illustrative schematics and figures by enabling quick identification of material–stimulation pairings best suited for specific clinical contexts in alveolar bone regeneration.

### 3.1 | Electroactive SSMs and Electric Field Regulated Pathways

Bone is a composite material composed of hierarchically arranged collagen nanofibrils, hydroxyapatite crystals, and cells [122]. Bone bioelectrical properties comprise dielectric, piezoelectric, pyroelectric, and ferroelectric properties [123]. Electrically active SSMs generate and transfer electrons or ions, exhibiting shape or volume change, when subjected to external electric field [124]. These SSMs are composed of piezoelectric materials (e.g., poly(vinylidene fluoride) (PVDF), poly-L-lactic acid (PLLA), polyhydroxybutyrate (PHB), polyhydroxybutyrate-co-valerate (PHBV)), piezoceramic materials (e.g., barium titanium oxide, bismuth sodium titanate, potassium sodium niobate, bioactive glass), conductive polymers (e.g., poly(3,4-ethylenedioxythiophene) (PEDOT), polyaniline (PANI), sulfonated/fluorinated polymers), organic materials (e.g., carbon nanotubes, fullerenes, graphene, quantum dots) and metals & metal alloys (e.g., Ag, Au, Ti, Sr). Piezoelectric biomaterials can generate electrical signals when subjected to mechanical stress or physical deformation (direct piezoelectric effect) and *vice-versa* (reverse piezoelectric effect). Piezoelectric ceramics, a type of piezoelectric material, are non-toxic, polycrystalline and ferroelectric biomaterials that produces spontaneous electric polarization against mechanical stress due to their internal electric dipole alignment [125]. In addition, some polymers allow transfer of electrons along their carbon skeleton due to the presence of conjugate bonds (alternating *σ* and *π* bonds) in their backbones, which are referred as conducting polymers [126]. These polymers can be used as pH- and/or electro-responsive, showing high electrical conductivity under physiological conditions. Metals and metal alloys are good conductors of electricity due to mobility of free valence electrons between the atoms [127]. Further, ionic biomaterials (such as ionic liquids, salts) also conduct due to the presence of ionic groups in their backbone and electrolytes in the biological environment [124]. These SSMs can be easily processed into porous hydrogels, fibers, films and lyophilized sponges or incorporated into other non-conductive polymers as conductive fillers in the composites. SSMs offer several advantages including synthetic flexibility, ability to enhance the mechanical strength, intrinsic conductivity and bioinertness, making them ideal for a variety of biomedical applications such as drug delivery, biosensors and tissue repair [128].

An exogenous electric current (ideally 5–100 μA [129]) applied to electroactive materials regulated osteoblastogenesis, osteoclastogenesis, angiogenesis, immunomodulation and anti-microbial effects through various downstream signaling pathways [111]. Recent research has demonstrated that ES induces anodic migration of macrophages and cathodic migration of monocytes, facilitating the localization and activation of these cells and promoting bone healing. Additionally, ES significantly enhanced macrophage phagocytosis and selectively regulated cytokine production, such as TNF-α and neurotrophin-3 [111]. ES using these electroactive SSMs repel the recruitment of inflammatory cells, facilitating the transition of macrophages from the proinflammatory M1 to the pro-healing M2 phenotype and reducing the secretion of proinflammatory cytokines at the defect site [130]. Hence, ES are predominantly used to treat several tissue injury types—e.g., skin wounds—enhance wound closure rates and reduce scar tissue formation [131] and crush sciatic nerve injuries—led to complete functional recovery [101]. ES has been shown to influence calcium channel activity, crucial in regulating cellular functions during bone remodeling. Activation of voltage-gated calcium channels in the cell membrane by ES causes an increase in the intracellular calcium ions, which binds to the activated complex comprising calmodulin (CaM, a Ca^2+^-binding protein), calcineurin (Cn) and nuclear factor of activated T cells (NFAT) transcription factors. This CaM/Cn/NFAT complex interactions mediated by Ca^2+^ influx promote proliferation and differentiation of osteoblast precursors and osteoblast cells with significant expression of markers such as BMP-2, alkaline phosphatase (ALP), collagen I, runt-related transcription factor 2 (RUNX2), osterix (OSTX), osteopontin (OPN), osteonectin (ON), bone sialoprotein (BSP) and bone gamma-carboxyglutamate protein (BGLAP) [129]. Osteoblast–osteoclast interactions are central to the early stages of bone healing, as they physiologically balance the processes of bone resorption and formation. Activating osteoclasts in the early stages of bone remodeling is beneficial for removing and absorbing damaged bone tissue [132]. Increased calcium channel activity induced by ES has been observed to enhance osteoclast function, thereby promoting osteoclast-mediated bone resorption. This suggests that electrical stimulation can facilitate osteoclast activity, which may influence the dynamics of bone remodeling during the initial phase of fracture healing [133]. Collectively, ES can impact macrophages and other immune cells in promoting regeneration.

Since bone is a piezoelectric material, applied ES mimics natural bioelectrical signals, thereby stimulating osteogenesis and increasing growth factor expression, such as BMPs and IGF, which drive bone regeneration [53]. ES also induces conformational changes in cytoskeletal proteins, redistribution and localization of focal adhesion receptors such as integrins and cadherins and activation of focal adhesion kinase (FAK) [134], activating osteogenic signaling pathways such as Ras, p38 kinase, PI3K/Akt, JNK, NF-κB, and MAPK/ERK pathways [130]. In addition, ES plays a key role in vascularization and angiogenesis in bone regeneration with increased VEGF expression. In tissue healing, ES has been observed to increase growth factor secretion, enhancing vascular growth and extracellular matrix synthesis, though the precise molecular mechanisms remain unclear [100, 101]. Further, these materials elicit anti-bacterial effects by increasing the membrane permeability through disruption of Na^+^/K^+^ pump, forming pores by developing stress in the bacterial cell wall and producing reactive oxygen species (ROS) to disturb the bacterial respiratory cycle [135]. These mechanisms discussed above were validated where an external and in situ ES mediated conductive and piezoelectric SSMs were used [136–138]. Though SSMs offer several advantages, they also present challenges. These materials need to address biodegradation issues, including achieving 100% renal clearance, cytotoxicity and immunological concerns. Additionally, their increased hydrophobicity and poor cell-material interactions, due to the absence of cell-adhesive motifs, pose limitations. This is particularly relevant for CNTs and graphene-based nanomaterials in physiological environments.

### 3.2 | Magnetoactive SSMs and Magnetic Field Stimulated Pathways

Magnetically active SSMs are usually made of pure metals, metal oxides, coated metallic and metal oxides in micro/nano-sized particles or wires. They are incorporated in composite matrices such as films, hydrogels and fibers [139]. Magnetic metallic particles show higher magnetic moments than magnetic oxide-based particles; however, they are highly oxidized in physiological environments. Hence, they are coated with protective surfactants or polymeric layers to prevent oxidation without compromising their magnetic properties [140]. Some of the commonly used ferromagnetic materials include Fe_3_O_4_ and γ-Fe_2_O_3_ in superparamagnetic form in addition to Co-and Ni-based alloys [141]. The external magnetic field can be applied in two ways: static magnetic fields (SMFs) and pulsed magnetic fields (PMFs). SMFs are constant magnetic fields produced by permanent magnets, classified by intensity as ultra-weak (5 μT–1 mT), weak (1 mT), moderate (1 mT–1 T), strong (1–5 T), and ultra-strong (> 5 T) [41]. Moderate-intensity SMFs are particularly explored for their ease of implementation in therapy. PMFs, in contrast, are designed to fluctuate at specific frequencies and intensities and can create both magnetic fields and electric currents [142–145]. Specialized devices enable PMFs by controlling the pulses’ frequency, amplitude, and duration [146–149].

When an external magnetic field (static or pulsed) is applied to magnetically active composite scaffolds, the magnetic particles undergo uniform directional alignment parallel to the magnetic field [150], causing an increase in the solution’s viscosity and mechanical stiffness of the scaffolds, which in turn regulate osteogenic differentiation. Higher matrix stiffness enables the differentiation of stem cells into osteoblasts and also activates various signaling pathways such as extracellular signal-regulated kinase (ERK), phosphatidylinositol 3-kinase (PI3K)/Akt, Wnt, and BMP pathways via integrin receptors [151]. Particularly, Wnt molecules are a group of secreted proteins that play a crucial role in various cellular processes, particularly in osteogenesis [152]. Key members of the Wnt family, including Wnt1, Wnt3a, Wnt4, Wnt5, Wnt10b, and Wnt13, are involved in bone formation. These Wnt proteins activate signaling pathways by binding to specific membrane receptors, such as Fzd1, Fzd2, Fzd4, and Fzd5, along with co-receptors Lrp5 and Lrp6. This binding triggers the canonical Wnt signaling pathway, leading to the accumulation of β-catenin in the cytoplasm and its translocation into the nucleus. In the absence of Wnt signaling, β-catenin is phosphorylated by the GSK3β kinase and subsequently degraded. However, when Wnts bind to their receptors, intracellular proteins like disheveled (Dsh) and axin are recruited to the membrane, preventing the degradation of β-catenin. As a result, β-catenin accumulates and enters the nucleus, where it interacts with the transcriptional repressor LEF/TCF. This interaction facilitates the recruitment of histone acetylase proteins, such as CBP/p300, leading to the activation of gene transcription mechanisms [152]. In addition, higher expression of collagen type I and osteogenic-specific genes such as runt-related transcription factor 2 (Runx2), Yes-associated protein (YAP), osteopontin (OPN), osteocalcin (OCN), and β-catenin was observed, confirming mineralization, osteoinduction, and osteogenesis [153]. It also alters the cell morphology by influencing the cytoskeleton proteins and intercellular interactions, allowing cells to attach and spread out more during osteogenic differentiation [151]. Upon removal of the magnetic field, these materials return to their original state, thereby allowing a fast and reversible change of the material. The use of sterile magnets in cell culture is simple to provide appropriate magnetic strength to alter the cell regulatory activities [154].

### 3.3 | Ultrasound-Active SSMs and Associated Signaling Pathways

Ultrasound waves are periodic high-frequency waves (> 20 kHz), which have some remarkable advantages such as non-ionizing nature, non-invasiveness, spatiotemporal controllability, deep tissue penetration capability, and economical. When ultrasound energy is propagated in a physiological medium, it produces thermal and non-thermal effects simultaneously [155]. The ultrasound generates an increase in temperature locally as the medium (tissues) absorbs ultrasound waves. Non-thermal effects include cavitation, vibrations, scattering, radiation force, microstreaming, and acoustic streaming [156, 157]. Cavitation effects are predominantly studied, resulting in the formation of irregular gas or vapor-filled bubbles (cavities) in the tissues. These bubbles oscillate and undergo repeated compression–rarefaction phases due to rapid changes in the pressure between the medium and bubble [157]. Common uses of ultrasound energy in biomedical applications include bioimaging, delivering target molecules across biological barriers, treating deep-seated tumors, and drug diffusion studies from nanoparticles, to name a few [158, 159]. However, higher intensity, frequency, duration, and interaction of ultrasound waves with tissues may produce hazardous effects. High-intensity ultrasound (5000–25,000 mW/cm^2^) creates high thermal energy and damages the bone tissues, leading to necrosis and fibrous tissue formation [160].

Low-intensity ultrasound has a positive impact in quickening bone repair and shortening the healing durations [161]. Low-intensity pulsed ultrasound (LIPUS) is a US FDA-approved non-invasive therapy, show non-thermal effects and support the acceleration of healing process in bone fractures, delayed unions and non-unions by enhancing osteogenesis, callus formation and bone mineral density and vasculature [116]. According to Wolff’s Law, LIPUS can cause micromechanical stimulation of the bone, resulting in osteogenesis [162]. In particular, the differential absorption of LIPUS may produce a gradient of mechanical strain in the healing callus, stimulating periosteal bone growth [163, 164]. Generally, these waves transmit mechanical stress akin to shear stress, stimulating osteoblast precursors to release growth factors like BMP, TGF-β, and VEGF, further enhancing bone regeneration [99, 165]. In osteogenesis, surface charges can absorb beneficial proteins, form an ECM layer, and promote cell deposition and tissue remodeling while simultaneously activating multiple molecular transduction mechanisms, such as calcium signaling, Wnt/β-catenin pathway, TGF-β/BMP, and MAPK/ERK, to induce osteogenesis [166].

Among FDA-approved non-implantable bone growth stimulators, “Exogen Ultrasound Bone Growth Stimulator (EXOGEN)” uses LIPUS with the following US parameters—power density: 30 mW/cm^2^, frequency: 1.5 MHz, pulse repetition rate: 1 kHz, pulse width: 200 μs, radiating area: 3.88 cm^2^, and temporal average power:177 mW. LIPUS shows an increased effect in the early (inflammation and callus formation) phases of bone healing however show minimal effect in the remodeling phase of fracture healing. During the inflammatory and callus formation phases of bone healing, ultrasonic waves demonstrate anti-inflammatory property by upregulating the expression of proinflammatory and inflammatory cytokines (e.g., IL-6, IL-8, IL-10, TNF-α) [167] and accelerates new blood vessel formation with high VEGF expression and ossification process at the defect site [168]. LIPUS creates mechanical force in the form of sound waves and act on osteoblasts to differentiate through Hedgehog, FAK, integrin/PI3K/Akt and ERK signaling pathways that are responsible for cell adhesion, survival, migration and proliferation [116, 169]. It can also regulate the expression of ALP, osteogenic transcription factors such as Runx2 and OSX and translation of osteogenic proteins such as cyclooxygenase-2 and prostaglandin E_2_ [170]. Other FDA-approved US-based devices uses low electrical or electromagnetic fields to provide mechanical stress in the bone defect site, allowing formation and differentiation of osteoblasts and osteoclasts with bone resorption ability [171].

Some of the commonly used ultrasound-responsive materials include piezoelectric ceramics (barium titanate (BaTiO_3_), zinc oxide (ZnO), lead zirconate titanate (Pb(Zr_x_Ti_1−x_)O_3_), calcium titanate (CaTiO_3_), boron nitrate (B(NO_3_)_3_) and hydroxyapatite (Ca_10_(PO_4_)_6_(OH)_2_)), micellar particles, nanodroplets, microbubbles and liposomes. These materials were incorporated or coated in scaffolds made of polymers and Ti alloys, providing stress-generated potentials (SGPs) like native bones. For instance, the coating of BaTiO_3_, a piezoelectric ceramic, over Ti6Al4V implants improved the implant’s porosity and surface hydrophilicity. These implants, when subjected to LIPUS, generate microcurrents upon mechanical deformation and showed better cell regulatory functions with bone marrow mesenchymal stem cells in vitro than non-coated Ti implants. In vivo implantation of porous Ti-based implants in large segmental bone defects for 12 weeks had enhanced new bone formation with better osteogenesis and osseointegration, promoting deposition of Ca^2+^ ions or salts at the site of bone defects [117].

### 3.4 | Mechanoactive SSMs and Mechanotransduction Pathways

Mechanoactive smart biomaterials respond by altering their mechanical properties in response to intrinsic or extrinsic mechanical deformation. When mechanical stimulus is applied to these materials, they deform or disintegrate to deliver the target molecules (e.g., drugs, growth factors), and provide mechanical cues (e.g., high tensile and compressive strength) for appropriate remodeling environment to repair and regeneration, thereby maintaining the body homeostasis. Biocompatible natural and synthetic polymers such as fibrin, collagen fibers, phenylboronic acid crosslinked 4-arm PEG-based, carboxymethylcellulose/phenylboronic acid-based and hydrazone-based hydrogels display strain-stiffening property, which is also a common characteristic of biological tissues such as bone. The collagen fibers present in bone ECM interlock tightly and orient themselves parallel to the applied direction of stress, leading to increased bone stiffness. Like bone, other tissues such as skin and blood vessels also display stiffening behavior under stress/strain (e.g., stretch), allowing them to prevent fracture or rupture beyond a certain stress level. Both natural and synthetic-based crosslinked polymers exhibit non-linear strain-stiffening behavior, where they become stiffer beyond the critical load/stress limit. This behavior is observed in biopolymer networks, which primarily depends on the type of physical (primary or secondary bonds) interactions involved in entanglements in addition to the polymeric network structure and their properties. This was attributed by two basic mechanisms—bending and stretching-dominated. The former is due to pulling out whereas the latter is due to axial reorientation of the fiber networks along the stress path [118]. Mechanical loading also induces osteoblastic precursor proliferation through the secretion of growth factors like BMPs and IGF, promoting bone formation [96, 172]. The underlying mechanisms of mechanical stimulation to bone regeneration are not yet well understood. However, studies reported that the mechanical stimulus increase ALP activity, mesenchymal stem cell differentiation to osteoblasts, upregulate the expression of osteogenic protein and genes (osteopontin, osteocalcin, collagen I) and activate potent downstream signaling pathways such as FAK/MAPK to regulate osteogenesis.

Mechanoactive smart biomaterials respond to mechanical forces—either intrinsic or externally applied—by adjusting their mechanical properties, releasing bioactive molecules, or delivering direct mechanical signals that promote bone repair and remodeling [173]. Beyond general mechanobiology, recent research has identified specific mechanosensors that link these mechanical cues to intracellular signaling pathways. Piezo1 and Piezo2 are membrane-spanning ion channels that open in response to tensile or compressive forces, enabling rapid Ca^2+^ influx. This calcium entry activates the Ca^2+^–calmodulin–calcineurin (CaM–Cn) complex, which in turn triggers NFAT transcription factors to promote expression of osteogenic genes such as Runx2, alkaline phosphatase (ALP), and osteocalcin [174]. Similarly, TRPV4 channels, sensitive to osmotic and shear stress from cyclic loading or scaffold deformation, initiate MAPK/ERK and PI3K/Akt signaling cascades that enhance osteoblast proliferation, matrix formation, and mineralization [175]. Mechanical signals are also transduced through the integrin–cytoskeleton axis. Mechanical deformation of scaffolds induces integrin clustering at focal adhesions, where adaptor proteins like talin, vinculin, and filamin transmit forces to the actin cytoskeleton [176]. This activates focal adhesion kinase (FAK) and Src kinases, leading to downstream RhoA/ROCK signaling that regulates cytoskeletal tension and nuclear localization of YAP/TAZ co-activators—key modulators of stem cell differentiation toward osteogenesis [177]. Mechanoactive SSMs—such as strain-stiffening collagen or PEG-based hydrogels and load-bearing polymer–ceramic composites—leverage these pathways by tuning their stiffness and viscoelastic properties to mimic native bone extracellular matrix. Under cyclic loading, these scaffolds upregulate Piezo1/2 and TRPV4 expression, boost ALP activity, and increase osteocalcin production compared to static conditions. In vivo studies of mechanoactive scaffolds implanted in alveolar bone defects show increased bone volume and improved integration, underscoring their promise for mechanically dynamic craniofacial applications [174, 175].

### 3.5 | Combination Therapies

Healing of critical-sized bone defects and delayed non-unions remain an unresolved challenge while using conventional orthopedic and trauma implants. By integrating physical stimulation methods such as pulsed electromagnetic field (PEMF) with the commercially available implants (e.g., bone screws/plates [121]), these implants and their procedures can be more functional, convenient, and on-demand to accelerate the bone healing process locally in the fracture site. Some of the common biological therapeutics use for bone healing include the use/delivery of pharmacological drugs (e.g., pamidronate, alendronate, zoledronic acid), hormones (e.g., adrenomedullin, melatonin, parathyroid hormone), growth factors (e.g., BMPs, TGF-β), cells (e.g., MSCs, peripheral blood stem cells) and peptides (e.g., calmodulin-gene related peptide). These biological therapies can also be combined with physical stimulus (dual/multiple) to target multiple stages in bone remodeling for synergistic stimulation of bone growth, healing and reduction of bone breakdown in the fracture site. This might be beneficial for patients with complex bone injuries and those who do not respond to single treatment. However, it is crucial to monitor patients as they may also experience negative effects of each treatment. Among several options for synergistic multiple treatments, combining physical stimulation and delivery of pharmacological drugs are safe, cost-effective and non-invasive with controlled ease of access to improve bone tissue healing and remodeling [178]. Here, optimal drug dosage, stimuli parameters and delivery duration of drugs/stimuli needs to be considered during the procedure. Such combination therapies are studied for femur, tibia, and radial bone defects [119–121] in preclinical models, thus necessitating a detailed investigation for treating craniofacial, maxillary and mandibular defects.

### 3.6 | Immunomodulatory Effects of SSMs in Alveolar Bone Regeneration

The immune microenvironment, particularly the dynamic polarization of macrophages between pro-inflammatory (M1) and pro-healing (M2) phenotypes, is critical for successful bone regeneration. Table 4 overviews the immunomodulatory effects of SSMs in regulating the inflammatory cytokines and growth factors during alveolar bone regeneration. These SSMs are designed to actively modulate this process, effectively acting as “immune-instructive” scaffolds to improve healing outcomes.

### 3.7 | Degradation, Byproduct Toxicity, and Clearance Considerations in SSMs

The degradation behavior of smart stimuli responsive materials (SSMs) plays a critical role in determining their safety, immunological response, and clinical translatability (Table 5). Biodegradable SSMs—particularly natural polymers and aliphatic polyesters—undergo degradation through hydrolytic, enzymatic, mechanical, or combined mechanisms. Naturally derived polymers typically degrade slowly and yield biologically familiar byproducts (e.g., sugars, amino acids), resulting in high biocompatibility and efficient metabolic clearance. In contrast, synthetic polyesters degrade into acidic products that may lower local pH and trigger inflammatory responses, sterile abscess formation, or foreign body reactions, depending on implant size, degradation rate, anatomical site, and buffering capacity of surrounding tissues.

Non-degradable and biostable polymers, including PMMA, PEEK, PEKK, and several conductive polymers, exhibit minimal degradation and are designed for long-term structural stability. These materials generally present low acute toxicity but may induce fibrous capsule formation and remain permanently in the body. Similarly, electroactive polymers (e.g., PVDF, PEDOT, PANI), carbon-based fillers, and many magneto and ultrasound-responsive materials display limited or no biodegradability, raising concerns for transient tissue regeneration applications that ideally require complete material clearance after healing. Composite SSM systems incorporating metallic, carbon-based, magnetic, or piezoceramic components introduce additional complexity, as these fillers may persist, accumulate in tissues, or rely on slow macrophage-mediated clearance. Collectively, these degradation and clearance challenges contribute to the predominantly preclinical status of many SSM technologies and highlight the need for resorbable, immunologically balanced platforms to enable clinical translation in alveolar bone regeneration.

## 4 | In Vitro and In Vivo Applications of Physical Stimulation in Alveolar Bone Regeneration

Alveolar bone injuries and associated healing outcomes significantly differ from healthy bone with inferior bone quality. The complex interactions between different cell types and healthy bone extracellular matrix (ECM) create a dynamic environment to promote constant bone remodeling in response to external stimuli and biomechanical load [181]. Bone is a piezoelectric material, meaning it generates electrical charges in response to mechanical deformation [182]. External stimuli, such as mechanical loads from daily physical activities, create piezoelectric effects in bone, promoting various cellular activities crucial for bone health and remodeling [183]. This understanding opens a world of possibilities for bone regeneration strategies, considering the role of biomechanical and external stimuli. These stimuli can create novel strategies for tissue engineering, enhance bone healing and complement the standard techniques used in clinics. The major external stimuli that can be applied to regenerate tissues are electrical, magnetic, ultrasound, and mechanical loading [183]. Often, these stimulation methodologies can be applied externally in conjunction with the other standard treatment modalities to enhance bone healing outcomes and quality further. However, these physical therapy modalities often result in varied bone healing outcomes due to a lack of detailed understanding of the mechanisms and inadequate stimulation at the site [184]. Developing methodologies with well-defined tissue engineering strategies can address this unmet clinical need. Table 6 summarizes the optimized physical stimulation parameters to treat alveolar bone defects in various in vitro & in vivo models.

### 4.1 | Electrical Stimulation

The piezoelectric effect is a critical component of the endogenous electric field, promoting bone healing and homeostasis. Therefore, ES is being explored as an adjunct technology in enhancing bone regeneration. The bone is electronegative in nature, which becomes significantly higher following bone injury or fracture [199]. Such an increase in electronegativity results in ionic current flow to the injury site, enhancing the flow of nutrients, growth factors, cell migration, and other biological processes that promote bone healing [200]. The attraction of Ca^2+^ ions to the negatively charged bone surface contributes to cell adhesion due to protein adsorption. The increased electronegativity also promotes osteoblast activity, enhancing ECM synthesis and mineralization at the injury site [201–204] Therefore, ES can accelerate bone healing by highlighting the electronegativity at the injury site. Studies have shown that placing the electrode on the injury site leads to faster and thicker callus formation [205].

**FIGURE 5 |:**
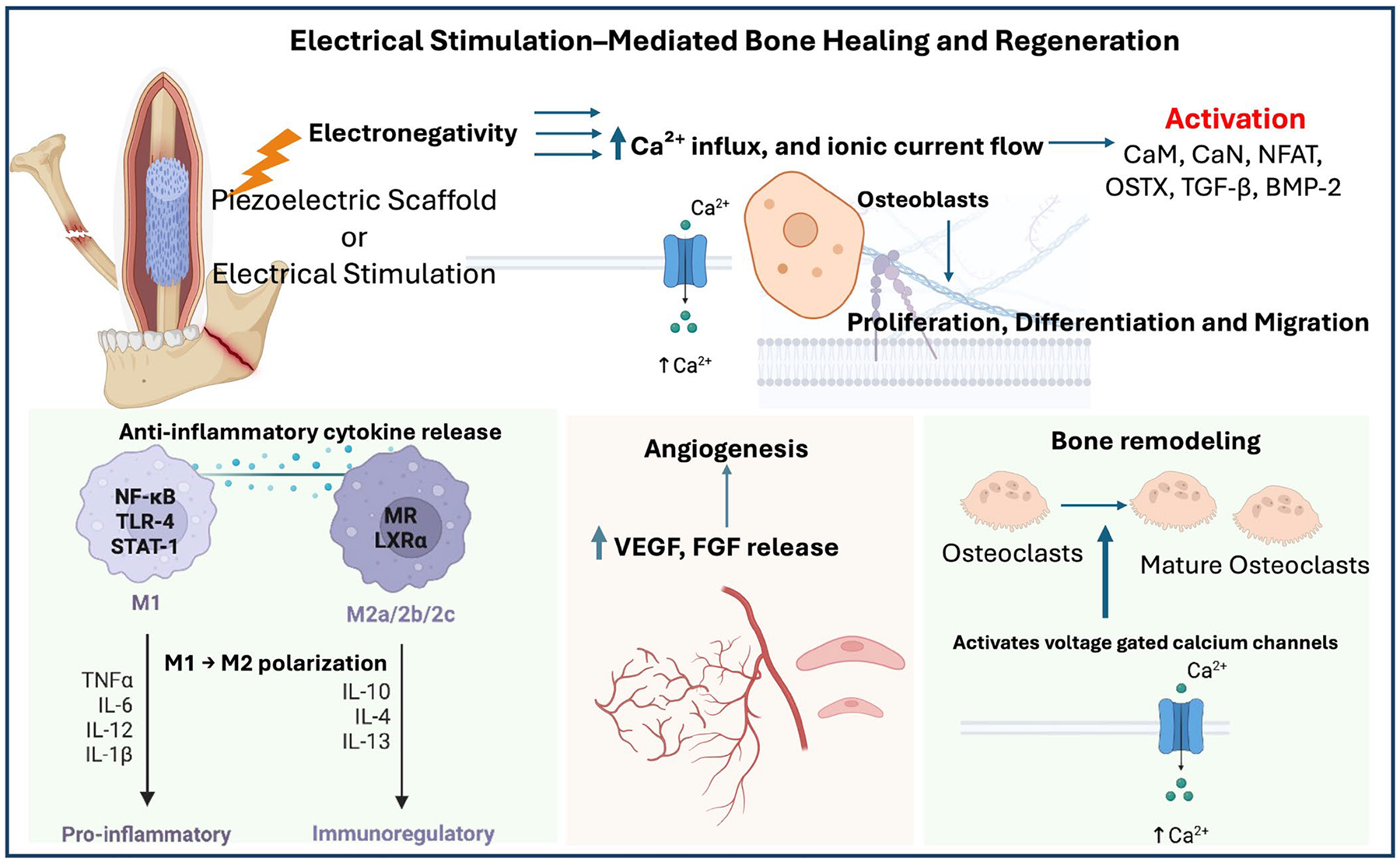
Electrical stimulation enhances bone regeneration through multiple coordinated mechanisms. ES increases bone surface electronegativity at injury sites, attracting Ca^2+^ ions and enhancing nutrient/growth factor flow. This promotes osteoblast proliferation, differentiation, ECM synthesis, and mineralization via CaM/CaN/NFAT, PKC, and β-catenin/E-cadherin signaling. Endothelial cells increase angiogenesis through VEGF and FGF release. Macrophages shift from pro-inflammatory (M1) to pro-healing (M2) phenotypes, secreting osteogenic cytokines while modulating inflammation via PI3K–AKT and RhoA/ROCK pathways. Facilitates the migration of both osteoblasts and macrophages, facilitating bone healing. ES activates voltage gated calcium channels promoting the differentiation of osteoclasts into active mature osteoclasts facilitating bone remodeling. Together, these effects accelerate healing, vascularization, and functional bone remodeling. Created with BioRender.com.

ES in bone healing offers two key benefits: enhancing osteoblast activity and promoting vascularization through the secretion of multiple osteogenic and angiogenic factors by resident cells, which are crucial for bone tissue engineering [206]. To date, extensive research in vitro has revealed how electrical stimulation affects the cellular activities of bone tissue lineages such as MSCs, osteoblasts, osteocytes, and osteoclasts. Electrical stimuli influence several biological processes involved in bone repair and regeneration, including cell proliferation [207, 208], apoptosis [209], alignment [210, 211], migration [212], adhesion [213, 214], and differentiation [112, 215] (Figure 5). Studies have shown that negative charges attract MSCs and inhibit the migration of osteoclasts [179, 216]. This process may promote osteogenesis at bone defect sites, supporting the previously mentioned hypothesis. ES also promotes the influx of Ca^2+^ into the cytosol of MSCs and osteoblasts by activating voltage-gated Ca^2+^ channels [137] and Cx43 [217]. This triggers pro-osteogenic signaling pathways like Calmodulin/Calcineurin/NFAT [129, 218] and PKC [138].

In contrast, osteoclasts (bone resorption) are inhibited by Ca^2+^ influx due to the activation of voltage-gated ion channels by electrical stimuli [219]. Other signaling pathways crucial for bone formation, such as β-catenin/E-Cadherin [220], inducible Nitric Oxide Synthase (iNOS) [221], and EphrinB2-EphB4 [222], are also activated by ES. Applied ES reduces inflammation through the modification of macrophage polarization and promotes bone healing. A study demonstrated that implanting an electroactive BaTiO_3_/poly(vinylidene fluoride-trifluoroethylene) nanocomposite membrane in diabetic rats accelerated bone repair [223]. This membrane promoted the shift of macrophages from a pro-inflammatory to a pro-healing phenotype by downregulating AKT2 and IRF5 within the PI3K-AKT signaling pathway. Similarly, diabetic rat periodontal bone defects reconstruction using an electroactive scaffold resulted in enhanced bone healing by facilitating macrophage polarization from the M1 to M2 phenotype via modulating glycolytic and RhoA/ROCK pathways [224]. The use of piezoelectric scaffolds in reconstructing bone defects can benefit from the mechanical deformation of the scaffold that generates microcurrent, eliminating the need for an external source to apply ES or implant electrodes [225]. A wireless system utilizing piezoelectric stimulation was developed with a piezoelectric hydrogel incorporating BaTiO_3_ nanoparticles. This system effectively reprogrammed macrophages toward the anti-inflammatory M2 phenotype, which in turn promoted the osteogenic differentiation of bone marrow mesenchymal stem cells, ultimately enhancing alveolar bone regeneration [226]. The efficacy of ES was tested in beagle dogs with 4-mm intrabony mandibular defects. Gold electrodes were attached to stone casts of the mandibles and fixed to the animals’ mouths. The experimental side received capacitively coupled electrical field (CCEF) stimulation (sinusoidal wave, 60 kHz, 5 V peak-to-peak) for 14 h daily. Histopathological analysis of decalcified bone showed significantly higher new bone formation (59.6% ± 10.78%), increased cementum thickness (3.60 ± 1.17 mm), longer junctional epithelium (1.25 ± 0.15 mm), and more osteoclasts (4.70 ± 0.75) on the experimental side, indicating enhanced tissue regeneration with ES [227]. Studies show that combining electrical stimulation (ES) with rhBMP-2 significantly enhances bone regeneration in rabbit mandibular defects [206]. In the experiment, hBMSCs were combined with a collagen sponge and hydrogel, placed in a PCL outer box with an ES device. Three groups were tested: Group 1 with rhBMP-2 without ES, Group 2 with rhBMP-2 and ES, and Group 3 with direct rhBMP-2 injection and ES. Radiography, histology, and μCT results revealed that Groups 2 and 3 had increased bone formation, with Group 3 showing the most significant regeneration and higher bone volume and mineral density compared to Group 1. The combination of ES, hBMSCs, collagen sponge, hydrogel, and rhBMP-2 proved effective for regenerating large mandibular defects [206].

### 4.2 | Magnetic Stimulation for Alveolar Bone Regeneration

Magnetic stimulation via static and pulsed field has been shown to enhance osteogenesis, bone density, mineralization, and implant integration with host bone [115, 228]. SMFs have demonstrated effectiveness in promoting osteoblast-like cell proliferation, migration, orientation, and differentiation [193, 229, 230] and osteogenic differentiation of BMSCs [231]. Animal studies show moderate-intensity SMFs improve bone mineral density and recovery in various conditions, including surgical invasion, ischemic bones, adjuvant arthritic rats, ovariectomized rats, and bone grafts [232–235]. For example, SMFs ranging from 110, 180, and 250 mT exposure to 3D scaffolds seeded with MG63 cells resulted in significantly higher ALP activity and mineralization levels than controls without stimulation [236]. Moderate-intensity SMFs are also more effective in treating metabolic diseases [237].

PMFs, on the other hand, expedite fracture healing, stimulate osteogenesis, and improve BMD [238–240]. Extremely low frequency (ELF)-PEMFs enhance osteogenic gene expression in human alveolar bone-derived MSCs in vitro [185]. In primary rat calvaria osteoblasts, exposure to PMFs at 50 Hz and 0.6–3.6 mT for 90 min/day showed the highest stimulation of proliferation and osteogenic differentiation at 0.6 mT [241]. This stimulation is linked to increased cytosolic Ca^2+^ and calmodulin activation, both crucial for osteogenesis [242]. Treatments such as 15 Hz and 1 mT have induced osteogenesis in MSCs [58], and EMF-induced osteogenic effects are mediated by signaling pathways such as PKA and MAPK, leading to higher bone mineral apposition rates, quicker bone formation, and increased osteoblast numbers [125]. PMFs also promote osteogenesis by altering cell membrane permeability, cyclic guanosine monophosphate (cGMP), and cyclic adenosine monophosphate (cAMP) activity [243]. Additionally, PMF exposure over 4 weeks increased Wnt/Lrp5/β-catenin gene expression and indicated the involvement of the RANKL-RANK signaling pathway in osteoclastic development and activation [244]. PMFs are particularly effective in treating musculoskeletal disorders and improving nerve function recovery [237]. Despite the benefits, the effects of magnetic stimulation vary depending on intensity and frequency, even within the same field type, influencing intracellular iron content and other cellular processes [245]. This diversity in cellular responses highlights the need for further research to understand better the mechanisms of magnetic stimulation and optimize its therapeutic application for bone repair and regeneration.

Biomaterials that create magnetic fields facilitate magnetic stimulation of cells and tissues without external magnetic fields. By incorporating magnetic nanoparticles (MNPs) into their structure, such biomaterials and scaffolds for tissue reconstruction can be made [143, 246–248]. Typical MNPs are superparamagnetic iron oxide nanoparticles (SPIONs). Such SPIONs have been used in gene therapy, tissue engineering, hyperthermia, targeted imaging/drug administration, and cell tracking. SPIONs can be taken up, metabolized, and exocytosed by cells. SPIONs without exposure to a magnetic field have been shown to promote tissue healing [249], offer dynamic mechanical stimulations [250], encourage BMSCs osteogenic differentiation [251], and improve bone regeneration in vivo [252]. Bone defects in incisor sockets of Sprague–Dawley rats were treated with SPION-containing gelatin sponges, which improved bone regeneration compared to control gelatin sponges. Approximately 1.5-fold increase in bone volume per tissue volume (BV/TV) and BMD and angiogenesis were observed in gelatin sponges with SPIONs [252].

However, the current literature lacks systematic studies to show the benefits of magnetic biomaterials and their stimulation in promoting bone regeneration. Future research should prioritize three key areas: combining magnetically enhanced cells with magnetic scaffolds, exploring the biological effects of power frequency EMF alone and in combination with MNPs and scaffolds, and addressing the bio-safety concerns of magnetic strategies in bone tissue engineering, particularly given the short-term focus of current studies.

### 4.3 | Ultrasound Stimulation for Alveolar Bone Regeneration

In vitro studies revealed that LIPUS might improve cell proliferative activity and induce osteogenic differentiation [253]. It is reported that LIPUS has demonstrated accelerated mineralization in vitro with increases in osteocalcin, alkaline phosphatase, VEGF, and MMP-13 expression. Also, ultrasound signals have been proven to activate integrins, a family of mechanoreceptors found in many cells involved in fracture repair. After integrin activation, focal adhesions form on cell surfaces, activating various signaling pathways such as ERK, NF-κβ, and PI3 kinase [254]. These pathways have been directly related to the generation of COX-2 and prostaglandin, which are important in fracture healing processes such as mineralization and endochondral ossification [254]. Lipopolysaccharide (LPS) is a key factor in worsening periodontitis, and applying optimal LIPUS intensity to reduce LPS-induced inflammation has been elucidated [255]. Human periodontal ligament cells (hPDLCs) cultured from premolar tissue were treated with LPS for 24 h and then exposed to LIPUS at 10, 30, 60, and 90 mW/cm^2^ intensities. The goal was to identify the optimal intensity to inhibit the expression of inflammatory factors IL-6 and IL-8, which were measured using real-time PCR and ELISA [255]. LPS-induced inflammation suppressed osteogenic differentiation in hPDLCs. However, LIPUS at 90 mW/cm^2^ alleviated inflammation by inhibiting the NF-κB signaling pathway. This led to increased expression of osteogenic genes and enhanced osteogenic differentiation. Additionally, LIPUS has been shown to improve the therapeutic potential of BMSCs, further supporting its role in promoting bone healing [255].

### 4.4 | Mechanical Stimulation for Alveolar Bone Regeneration

Bone is a dynamic tissue whose growth and maintenance are significantly impacted by biomechanical stimuli generated by load-bearing activities. It has been established that mechanical cues play an important role in a variety of physiological processes [256, 257]. Periodontal ligament stem cells (PDLSCs) are sensitive to mechanical stress and can regulate physiological processes such as proliferation and differentiation by fine-tuning genes, signaling pathways, and ion channels [258, 259]. Therefore, it is essential to elucidate the adaptive changes and self-renewal capacity of PDLSCs under mechanical stimuli. Mechanical forces also cause bone matrix deformation, generating hydraulic pressure and interstitial fluid flow through voids and channels, including lacunae-canaliculi. This interstitial bone fluid flow facilitates nutrient, cytokine, and growth factor transfer, crucial for bone tissue remodeling during homeostasis, repair, and regeneration. Therefore, it is evident that interstitial bone fluid is a key regulator of bone mass and architecture in response to mechanical stimulation [260, 261]. Additionally, mechanical loading is known to increase osteoblastic cell proliferation, differentiation, ECM deposition, and cytokine/growth factor secretion [262]. Though it is understood that mechanical stimuli play a crucial role in bone tissue remodeling during homeostasis, repair, and regeneration, they cannot function independently and work in combination with the biophysical and biochemical microenvironment within bone tissue.

Bone tissue cells interact in a complex network, including osteocytes, osteoblasts, osteoclasts, MSCs, endothelial cells, and immune cells like neutrophils and macrophages. This involves a complex network where focal adhesions relay mechanical signals from ECM to cells, forming a direct link between the cytoskeleton and ECM. The transmission of mechanical stimuli by focal adhesions affects FA, which transmits mechanical stimuli that affect cytoskeleton arrangement and crosslinking, affecting cell adhesion, stretching, and migration. This activates key mechanotransduction pathways in osteogenesis regulation, including YAP/TAZ, MAPK/ERK/JNK, and RhoA/ROCK [263]. Furthermore, mechanical stimuli can activate ion channels like Piezo1/2 and TRPV4, causing a calcium ion influx. This activates various signaling pathways like Ca^2+^-calcineurin-NFAT1 and Wnt/β-catenin, which in turn regulates osteogenesis [264, 265]. The activation of ion channels (TRPV4) is linked to mechanosensitive primary cilium in bone tissue cells [266, 267] upon exposure to bone interstitial fluid flow shear stress, which is known to trigger the activation of TRPV4 ion, leading to a Ca^2+^ ion influx that enhances osteogenic differentiation of MSCs [268]. Studies show that mechanical stimuli significantly promote vascularization and angiogenesis during bone healing, which is crucial for bone repair and regeneration [269–271]. Besides oxygen and nutrient flow to the bone by interstitial fluid, increased angiogenesis in bone regeneration can alter osteoblastic functions by releasing endothelial-derived factors like endothelin and nitric oxide. These factors enhance osteoblast proliferation and suppress bone resorption by osteoclasts. Mechanical stimuli, such as mechanical loading of bone, can stimulate the release of pro-angiogenic factors like nephronectin, VEGF, HIF-1, EGFL, and Notch ligands, all crucial in regulating endothelial cell differentiation and proliferation [272–275].

Till date, most studies on mechanical stimulation’s effects on osteogenesis and bone repair are based on in vitro models, making it difficult to translate to in vivo animal studies or human clinical models. Although physiotherapy rehabilitation programs are prescribed to enhance bone healing by mechanical stimulation, no clinically relevant technology has yet been achieved. Current animal studies use external cyclic mechanical stimulation, which is intermittent and limited in therapeutic effects compared to exercise and physical activities.

## 5 | Translational Challenges and Future Directions of Physical Stimulation–Smart Biomaterials in Alveolar Bone Repair

The clinical application of smart stimuli-responsive materials combined with physical stimulation modalities for alveolar bone regeneration represents a rapidly evolving but still immature area of regenerative medicine. This field lies at the intersection of materials science, biomedical engineering, and clinical dentistry, and has gained increasing attention because of its potential to overcome limitations associated with conventional bone grafting approaches. Although many SSM platforms were conceptually developed years ago, a mechanistic understanding of how these materials regulate osteogenesis, angiogenesis, immunoregulation, and anti-apoptotic pathways at cellular and tissue levels has only recently emerged [276]. The appeal of SSMs lies in their ability to dynamically respond to environmental cues such as electrical, mechanical, magnetic, or ultrasonic signals using relatively simple and robust material designs that enable rapid responsiveness to external stimulation [276].

Despite these advantages, multiple biological, technical, and regulatory challenges continue to impede reliable clinical translation. One major limitation is the unpredictable and often non-specific material response under complex physiological conditions. Variations in local pH, enzyme activity, inflammatory milieu, and ionic composition can alter stimulus sensitivity, resulting in responses that are insufficient, delayed, or difficult to control for on-demand therapeutic applications [139, 277, 278]. These issues are exacerbated during extended implantation periods, where biodegradable scaffolds may experience progressive loss of mechanical integrity and diminished responsiveness as degradation proceeds. Additionally, early adsorption of proteins and adhesion of cells on scaffold surfaces can further dampen or alter stimulus transduction kinetics, particularly during the critical early stages of regeneration.

Another challenge arises when multiple stimuli or combined stimulation strategies are employed. Excessive sensitivity or cross-reactivity may lead to off-target or antagonistic effects, complicating therapeutic predictability. Furthermore, applied physical stimulation parameters such as intensity, amplitude, penetration depth, frequency, and duration are strongly influenced by defect size, anatomical location, tissue density, and patient-specific biological properties. As these variables differ widely among individuals, standardization of stimulation protocols remains difficult, necessitating careful patient-specific calibration to achieve consistent outcomes. The scarcity of large-scale, long-term preclinical and clinical datasets, coupled with challenges in reproducible manufacturing and non-standardized regulatory pathways, further constrains widespread clinical adoption of SSM-based therapies [139, 277, 278].

Clinically, alveolar bone defects are managed primarily using autografts, allografts, xenografts, and synthetic bone substitutes. Although widely adopted in orthopedic, dental, and maxillofacial practice, none of these approaches consistently achieves optimal outcomes across all indications. Autografts, while considered the gold standard because of their osteogenic capacity, are limited by donor site morbidity and restricted availability. Allografts and xenografts pose risks of immune response, infection, and reduced bioactivity resulting from processing, whereas synthetic grafts, despite advantages in availability and mechanical strength, lack intrinsic biological signaling and often result in suboptimal integration or compromised long-term compatibility. Collectively, these limitations highlight the need for advanced bioengineered alternatives that can deliver safer, more effective, and more predictable regenerative outcomes.

Clinical studies addressing alveolar bone defects associated with tooth extraction, congenital malformations, and craniofacial abnormalities have evaluated a range of treatment modalities, including biological grafts and grafts augmented with platelet-rich plasma (PRP), bone morphogenetic proteins (BMPs), and stem-cell-based interventions. Among these approaches, iliac cancellous bone autografting has demonstrated high success rates (~90%) with favorable outcomes such as enhanced bone density, improved ridge preservation, and reliable implant integration, particularly in cleft lip and palate patients [279]. Recombinant human BMP-2 and bovine-derived demineralized bone matrix have also yielded clinical outcomes comparable to autografts with respect to bone density, facial symmetry, ridge preservation, and sinus augmentation; however, the long-term safety and efficacy of these bioactive agents—including stem cells—remain inadequately defined [279]. To date, no bone graft or substitute consistently achieves 100% clinical success across diverse patient populations and defect scenarios.

Emerging strategies that integrate advanced scaffold fabrication techniques, smart stimuli-responsive materials, pre-and post-surgical computational design, and artificial intelligence (AI)-assisted planning may enable development of truly personalized alveolar bone grafts. Nevertheless, these approaches remain at an early developmental stage, and significant heterogeneity in study designs and outcome reporting highlights the continued need for rigorous, high-quality translational research.

Patient-specific biological and mechanical factors are increasingly recognized as critical determinants of therapeutic success. Age-related declines in healing capacity, systemic comorbidities such as diabetes or chronic inflammation, and local infection status often necessitate incorporation of immunomodulatory, antimicrobial, or pro-angiogenic functionalities into scaffold design. Defect size and anatomical location dictate requirements for scaffold porosity, degradation kinetics, and mechanical reinforcement—larger or load-bearing defects may necessitate composite or mechanically strengthened constructs, while smaller or confined alveolar defects may be better addressed with injectable or hydrogel-based systems. Physical stimulation regimens must likewise be tailored to tissue condition to promote osteogenesis without overstimulation. These considerations emphasize the need for adaptable and modular SSM platforms capable of personalized configuration.

**FIGURE 6 |:**
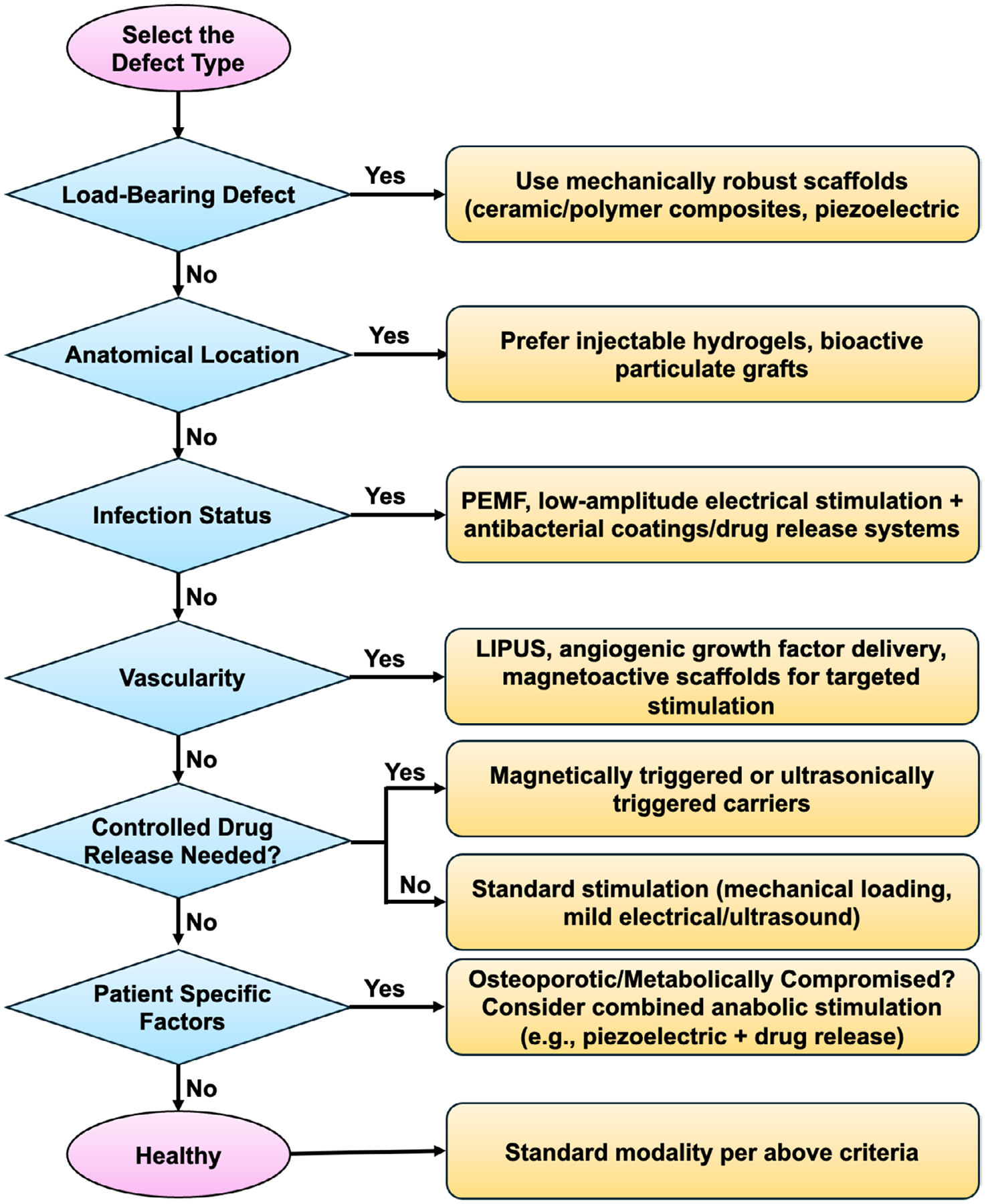
Clinical decision framework for selecting physical stimulation modalities and smart biomaterials.

Figure 6 presents a clinical decision-making framework that integrates defect characteristics, host factors, and therapeutic objectives to guide selection of physical stimulation modalities and smart biomaterials. This framework aligns scaffold composition with mechanical requirements (e.g., ceramic/polymer composites for load-bearing sites versus injectable hydrogels for confined defects) and incorporates infection status, vascularity, and drug-delivery needs to inform modality choice. By synthesizing preclinical and limited clinical evidence, this framework aims to enhance reproducibility and streamline translational decision-making (Figure 6).

Preclinical studies widely support the biological efficacy of physical stimulation–biomaterial combinations. Modalities such as low-intensity pulsed ultrasound (LIPUS), pulsed electromagnetic fields (PEMF), and piezoelectric scaffolds have consistently demonstrated increases in bone volume (BV/TV ~15%–45%) and mineral apposition rates (MAR ~20%–50%) in animal models [226, 280–282]. PEMF and LIPUS have achieved clinical acceptance in fracture-healing contexts, and limited pilot studies suggest potential applicability to alveolar defects [282–287]. However, translation into routine dental practice remains limited due to small, heterogeneous clinical trials and inconsistent outcome measures.

Table 7 summarizes the translational status of smart biomaterials and external physical stimuli investigated for bone regeneration. While some randomized controlled trials have reported improved graft consolidation, peri-implant bone density, or faster osseointegration with adjunctive stimulation, other studies have found no statistically significant benefit over conventional treatment [283, 285, 286]. Moreover, smart biomaterials capable of stimulus-responsive drug release remain largely preclinical, with no FDA-approved systems specifically indicated for alveolar bone regeneration. Instances of translational failure—such as BMP-loaded scaffolds combined with external stimulation failing to produce additive benefits—highlight unresolved questions regarding synergistic mechanisms and reproducibility [301]. Differences between small-animal, large-animal, and human healing dynamics further complicate translation [62, 63].

Material degradation behavior represents a major contributor to clinical and regulatory uncertainty. Biodegradable natural polymers generally exhibit favorable biocompatibility but suffer from poorly controlled degradation rates, whereas synthetic polyesters can generate acidic byproducts that induce localized inflammation, especially in confined oral environments. Conversely, non-degradable and electroactive materials, while mechanically stable, may persist long-term and provoke fibrous encapsulation or chronic inflammation. Composite systems incorporating carbon-based fillers or inorganic stimuli-responsive particles introduce additional biosafety and clearance concerns that remain insufficiently characterized.

From a regulatory standpoint, most SSM platforms are classified as combination products, substantially increasing approval complexity. Manufacturing reproducibility, long-term biocompatibility, degradation validation, and post-implant clearance remain key regulatory barriers. Table 8 details clinical setbacks and regulatory challenges associated with different SSM material categories, highlighting why not fully integrated SSM-based systems have yet achieved FDA approval for alveolar bone regeneration.

Looking forward, large-area bone regeneration continues to pose a substantial clinical and economic burden, with inadequate vascularization, mechanical mismatch, and poor remodeling contributing to non-union and compromised outcomes [302–308]. Although FDA-approved bone graft substitutes exist [107, 309–311], most lack intrinsic bioactivity and rely on adjunctive biological or mechanical cues to enhance performance. Growth-factor-based therapies, including BMPs and VEGF, have shown limited and inconsistent success in clinical trials due largely to dosing, delivery, and release kinetics challenges [306, 312–329]. Physical stimulation modalities—including mechanical loading, electrical stimulation, magnetic fields, and LIPUS—offer non-invasive means to enhance endogenous repair by stimulating resident cells to release osteogenic and angiogenic factors [99, 115, 165, 193, 201–204, 228–240, 253, 254, 258, 259]. The accessibility of mandibular and alveolar bones further supports their clinical feasibility [100, 101, 330]. Smart materials capable of transducing these signals locally, especially when integrated with advanced micro- and nanofabrication approaches, hold promise for overcoming current limitations [184, 331].

Finally, while combinatorial stimulation strategies are conceptually attractive, systematic evaluation of multi-modal physical stimulation remains sparse, particularly for alveolar bone regeneration. Future work must focus on standardized, controllable SSM-based platforms that permit precise tuning of stimulus type, magnitude, and timing to clarify synergistic versus antagonistic interactions. Such efforts will be essential to advance personalized, predictable, and durable solutions for alveolar bone regeneration.

## Figures and Tables

**TABLE 1 |:** Comparison of long bone and alveolar bone.

Feature	Long bone	Alveolar bone
Structure	Long, cylindrical shape with a central marrow cavity	Irregular shape with alveolar sockets
Primary function	Structural support, movement	Supports teeth, adapts to dental changes
Mechanical properties	Varies widely, high strength and moderate remodeling	High compressive strength in alveolar regions for chewing
Remodeling rate	Relatively slow	Rapidly remodels in response to local stimuli
Response to loss	Gradual loss due to systemic factors	Rapid loss due to tooth extraction or disease

**TABLE 2 |:** List of available additive manufacturing techniques involved in the fabrication of mandibular grafts.

Technique	Advantages	Disadvantages	Materials and examples of mandibular grafts
Selective laser sintering (SLS)	High resolution, complex geometries, no support structures	Time-consuming, discontinuous process, rough surface finish, post-processing required, uses higher laser power for printing process	Titanium SLS-printed part implanted in left mandible along with an iliac crest bone graft; Achieved reconstruction of mandibular discontinuity, restoration of mandibular symmetry with pleasing facial appearance [69] Endosseous blade implant made of titanium alloy (Ti-6Al-4 V) implanted in atrophied posterior mandible site; Supported proper prosthetic fixation with implant dislocation and stability after functional loading for up to 2 years [70] 3D printed PEEK prosthetics with screw holes was fabricated to secure the non-vascularized fibula autograft within the mandible; Provided good mechanical support, restored occlusal and aesthetic function for effective mandibular reconstruction [71]
Electron beam melting (EBM)	Excellent for metallic implants, high strength and density, biocompatible	Expensive equipment, limited to conductive materials, slow build process, time-consuming post-processing	Titanium-based patient-specific bridging plates were fabricated using EBM for left lateral mandibular defect associated with severe maxillary occlusal cant; Implant had adequate strength to withstand the biting force, recovery of masticatory function and face symmetry [72] Whole mandible Ti6Al4V 3D mesh scaffold was designed from patient’s CT images using CAD and fabricated using EBM; Reduction in the production & surgery time, exhibited good biocompatibility with no necrosis after 1 month post-operation [73]
Selective laser melting (SLM)	High precision and density (~99%), low porosity, high mechanical strength, suitable for complex structures	Requires extensive support structures, high operational costs, risk of residual stress	Titanium and cobalt-chromium implants for mandibular reconstruction Patient-specific functional implants (PSFI) were developed using SLM and implanted in 22 patients with fibula transplantation; No postoperative complications such as implant dislocation and wound healing disturbances were observed even after 6 months [74] Porous Ti6Al4V 3D-printed titanium mesh (SLM) implanted over mandibular defect together with bone autograft; Achieved stable space maintenance, enhanced bone regeneration, and improved defect healing due to high porosity and customized fit [75]
Fused deposition modeling (FDM)	Cost-effective, wide range of biocompatible thermoplastic polymers, easy to operate	Lower resolution, limited mechanical strength, post-processing required	Polycaprolactone (PCL)-based scaffolds, Polylactic acid (PLA) implants, Polyetherketonketone (PEKK) implants Gypsum mandibular model was replicated digitally and 3D printed using PEKK; Material showed superior tensile and bending strength similar to native human bone, emphasizing its potential for mastication and related stresses [76]
Digital light processing (DLP)	High surface finish and resolution, fast printing speeds, suitable for photopolymerizable biomaterials	Limited to photopolymers, fragility of printed structures, potential toxicity of resins	Hydrogel-based scaffolds, UV-curable polymer grafts DLP-based polymethyl methacrylate (PMMA)-based mandibular overdentures and maxillary complete dentures enabled negligible dimensional changes and water absorption compared to the conventional denture resins [77] DLP-printed bioactive glass (e.g., 45S5) scaffold with integrated porous architecture; Enhanced scaffold degradability and osteogenic properties [78] DLP-printed nanoscale calcium silicate (nCSi) bioceramic scaffold doped with Mg and GelMA/Si-HPMC hydrogel membrane implanted in periodontal defect model; Achieved hierarchical regeneration of bone and periodontal ligament, with improved tissue integration and functional repair [79]
Extrusion-based 3D bioprinting	Multi-material versatility, compatible to high cell density and high structural integrity	Complex material preparation; potential mismatch in degradation rates; processing challenges in uniform material distribution	Natural/synthetic polymers, inorganic materials GelMA/alginate/bioactive glass encapsulated with mesenchymal bone marrow stem cells were extrusion-bioprinted and implanted in mandibular premolar region in dogs; Achieved effective cell delivery, promoted periodontal tissue regeneration [80]

**TABLE 3 |:** Comparative summary of smart stimuli-responsive materials (SSMs) categorized by material type, stimulation modality, application site, biological outcomes, and translational status for alveolar bone regeneration.

Material type	Stimulation modality	Target application site	Observed biological outcomes	Translational status
Piezoelectric polymers (e.g., PVDF, PLLA, PHB, PHBV)	Electrical stimulation (ES) via piezoelectric effect	Alveolar bone defects; load-bearing regions	Generates microcurrents under stress; promotes osteoblast differentiation, Runx2, ALP, collagen I, osteopontin expression; angiogenesis via VEGF	Preclinical in rodents, canine mandibular defect models [112, 113]
Conductive polymers (e.g., PEDOT, PANI)	Direct ES	Periodontal and mandibular bone repair	Enhances osteoblast proliferation, macrophage polarization (M1 → M2), modulates inflammatory cytokines, promotes mineralization	Preclinical animal models; early-stage feasibility [114]
Metallic conductors (e.g., Au, Ag, Ti alloys)	ES	Mandibular segmental defects	Promotes osteogenesis and angiogenesis; antibacterial activity via ROS and membrane disruption	Clinical use in dental implants (Ti); functionalization for ES under study [64, 65, 88]
Magnetic nanoparticles (e.g., Fe_3_O_4_, γ-Fe_2_O_3_)	Static or pulsed magnetic fields	Critical-sized defects, alveolar ridge augmentation	Enhances osteogenic gene expression (Runx2, OPN, OCN, (β-catenin), collagen I deposition; modulates cytoskeleton alignment	Preclinical animal models [115]; limited human pilot studies
Ultrasound-active ceramics (e.g., BaTiO_3_-coated Ti6Al4V)	LIPUS	Large mandibular bone defects	Generates microcurrents; enhances osseointegration, osteogenesis, Ca^2+^ deposition; upregulates BMP, VEGF	FDA-approved LIPUS devices for fracture healing [116]; SSM-coated scaffolds in preclinical stage [117]
Mechanoactive hydrogels and composites (e.g., collagen, PEG-based strain-stiffening hydrogels)	Cyclic mechanical loading	Periodontal ligament and alveolar bone	Promotes MSC osteogenic differentiation, ALP activity, osteocalcin expression; mimics ECM stiffness adaptation	Preclinical studies; not yet clinically applied in alveolar bone [118]
Combination SSM systems (e.g., ES + rhBMP-2 loaded scaffolds)	Dual/multi-stimulus (electrical, magnetic, mechanical, ultrasound)	Critical-sized mandibular defects	Synergistic effects on bone volume, mineral density, and vascularization; reduced healing time	Preclinical large-animal studies; early translational interest [119–121]

**TABLE 4 |:** Immunomodulatory effects of smart stimuli-responsive materials (SSMs) in alveolar bone regeneration.

SSM type and modality	Macrophage polarization effect	Key cytokine/factor changes	Proposed mechanisms
Electroactive (ES, piezoelectric, conductive polymers) [179]	Promotes M1 → M2 transition	↓ TNF-α, IL-1β, IL-6; ↑ IL-10, TGF-β, NT-3	Ca^2^+ influx → PI3K/Akt, RhoA/ROCK; enhanced osteoblast–macrophage crosstalk
Magnetoactive (static/pulsed magnetic fields) [151, 153]	Skews toward M2 phenotype	↓ TNF-α, IL-12; ↑ VEGF, BMP-2, IL-10	Integrin–FAK signaling; β-catenin activation
Ultrasound-active (LIPUS, piezoelectric ceramics) [117, 168]	Reduces prolonged inflammation; supports M2 recruitment	↑ VEGF, BMP-2; modulates IL-6, IL-10	Integrin/FAK–MAPK/ERK activation; mechanical microstreaming
Mechanoactive (strain-stiffening hydrogels, composites) [118, 180]	Maintains regenerative M2 profile	↑ VEGF, PDGF; ↓ pro-inflammatory cytokines	ECM stiffness mimicry; cytoskeletal tension → YAP/TAZ signaling

**TABLE 5 |:** Degradation behavior, byproducts, and clearance mechanisms of major SSM classes.

SSM category	Materials used	Degradation rate	Degradation byproducts	Clearance/biological impact	Translational considerations
Natural biodegradable polymers	Collagen, chitosan, gelatin, silk fibroin, fibrin, hyaluronic acid, CMC	Slow–moderate	Sugars, amino acids, peptides	Enzymatic resorption; Metabolized via native pathways	High biocompatibility; clinically favorable
Synthetic biodegradable polyesters	PLA, PGA, PLGA, PCL, PLLA	Moderate–slow (composition-dependent)	Lactic/glycolic acid (acidic)	Local pH drop; inflammation risk depending on site/size	Requires buffering/composites
Biodegradable bio-polyesters	PHB, PHBV, PHAs	Slow	Hydroxy acids	Macrophage-mediated clearance	Promising; limited clinical data
Degradable PEG hydrogels	Crosslinked PEG systems	Tunable	Low-MW PEG fragments	Renal clearance	Favorable when fully degradable
Non-degradable structural polymers	PMMA, PEEK, PEKK	Non-degradable	None	Fibrous capsule formation; long-term persistence	Used when permanence acceptable
Conductive/electroactive polymers	PVDF, PEDOT, PANI	Non-degradable	None	Persistent materials; encapsulation risk	Undesirable for transient regeneration
Carbon-based composites	CNTs, graphene, fullerenes, quantum dots	Non-degradable	None	Partial clearance or tissue accumulation	Safety concerns limit translation
Magneto/ultrasound-active fillers	SPIONs, BaTiO3, piezoceramics	Non-degradable	None	Slow macrophage-mediated clearance	Preclinical; no FDA approval

**TABLE 6 |:** Optimized physical stimulation-based treatment strategies utilized in in vitro and in vivo models for mandibular defects.

Physical stimulus	Parameters	In vitro/in vivo model	Biological outcomes	References
Extremely Low Frequency Pulsed Electromagnetic Fields (EMF)	Frequency: 10, 50, 100 Hz for 10 min/day Magnetic field strength: 6 G	Human alveolar bone-derived mesenchymal stem cells (hABMSCs)	Exposure of high frequency (50 and 100 Hz) to cells showed increased hABMSCs proliferation after 4 days, mineralized nodules formation (by ALP staining) and elevated osteogenic markers (ALP, mineralization, OC) in EMF groups; elevated vinculin, vimentin, and CaM expression.	[185]
EMF-DC	1 h/day for 7 days; 10, 50, 100, 150 mV/mm.	DPSC	Reduced metabolic activity, ALP, collagen, and calcium deposition; upregulated bone-specific genes (OC, RUNX2, BSP, DMP1).	[186]
mES	2 h/day at an intermittent Regimen (stimulation time: 38.62 s and rest time: 110.46 s) for 3 days; mES of 0.5, 38 & 75.5 μA.	DPSC	Enhanced proliferation and OC expression under 38 μA stimulation	[187]
Pulsed EMF (PEMF)	16 Hz, with 7.8 V/m between electrodes, 4 h per day for 45 days	Hindlimb suspension rat model	Controlled bone loss induced by simulated microgravity	[188]
EMF	15 Hz, 1 mT, 4 h/day	BMSCs, critical-sized calvarial defect in rats	Enhanced proliferation and osteogenic differentiation capacity of BMSCs	[189]
PEMF	15 Hz; 2.4 mT; 2 h/day; 8 weeks	KK-Ay mouse	Resisted type 2 diabetes mellitus-associated bone deterioration	[190]
EMF	30/45 Hz, 1 mT, 8 h/day for 20 days	ADSCs	Increased osteogenic differentiation	[191]
Static magnetic field (SMF)	130 mT, 1 h/day for 5 days	hABMSCs	Osteogenic potential of NHGH derived from hABMSCs	[192]
SMF	160 mT—20 days	Rat calvaria cell	Promoting osteoblastic differentiation and activation	[193]
LIPUS	Pulsed:1000 Hz, central: 1.5 MHz carrier, 30–60 mW/cm^2^, for 10–30 min/day	RAW264 macrophage cells	Increased osteoclast number, TRAP activity, and cathepsin K expression with 30 mW/cm^2^	[194]
LIPUS	Pulsed:1 kHz, central: 1.5 MHz, 30–90 mW/cm^2^, 15 min/day for 7 days	Human periodontal ligament stem cells (hPDLSCs)	Enhanced viability, anti-inflammatory response, and osteogenic differentiation of hPDLSCs	[195]
LIPUS	Pulsed:1 kHz, central: 1.5 MHz, 30 mW/cm^2^, 20 min/day for 7–14 days	Monoculture: Osteoblasts and Osteoclasts Co-culture: Osteoblasts and osteoclasts	Enhanced osteogenesis and osteoblast–osteoclast interaction; minimal effect on osteoclasts in monoculture	[196]
LIPUS	Pulsed: 0 Hz (continuous), central: 1–1.5 MHz, 30–60 mW/cm^2^, 5–10 min/day, single or for 3 days	Human fetal osteoblast cells (hFOBs) and human periodontal ligament fibroblasts (hPLFs)	Maintained osteogenic activity and increased OPG expression in hPLFs, minimal effects on hFOBs	[197]
LIPUS	Pulsed: 1 kHz, central: 1.5 MHz, 30 mW/cm^2^, 10 min/day for 7 days	Mandible slice organ cultures (ex vivo)	Enhanced osteoclast activity, dentin and cementum formation, and PDL cellularity after LIPUS	[198]

**TABLE 7 |:** Translational status of external physical stimuli on bone regeneration.

Approach	Highest preclinical model	Key preclinical metrics	Clinical status	Representative outcomes
LIPUS [60, 288–291]	Canine, non-human primates	BV/TV ↑ 20%–35%, MAR ↑ 25%, BMD ↑ 15%	Pilot human trials	Ridge height gain ~1 mm, faster osseointegration
PEMF [282, 292–294]	Canine, non-human primates	BV/TV ↑ 15%–30%, improved mechanical strength	FDA-approved (fractures); pilot alveolar studies	ISQ ↑, bone fill ↑ 10%–15%
Smart biomaterial+peptide [295, 296]	Rabbit, canine	BMD ↑ 25%–50%, lamellar bone formation	Phase I–II	Variable; some non-inferior to autografts
Smart biomaterial + growth factor [289, 292, 293, 297–300]	Canine, non-human primates	BV/TV ↑ 30%–50%, MAR ↑ 20%	Phase II (mixed results)	Some failures due to inflammatory response

**TABLE 8 |:** Clinical setbacks and regulatory barriers in ssm-based alveolar bone regeneration.

SSM category	Materials	Degradation rate	Degradation byproducts	Clearance/biological impact	Translational considerations
Natural biodegradable polymers	Collagen, chitosan, gelatin, silk fibroin, fibrin, hyaluronic acid, CMC	Slow–moderate	Sugars, amino acids, peptides	Enzymatic resorption; metabolized via native pathways	High biocompatibility; clinically favorable
Synthetic biodegradable polyesters	PLA, PGA, PLGA, PCL, PLLA	Moderate–slow (composition-dependent)	Lactic/glycolic acid (acidic)	Local pH drops; inflammation risk depending on site/size	Requires buffering/composites
Biodegradable bio-polyesters	PHB, PHBV, PHAs	Slow	Hydroxy acids	Macrophage-mediated clearance	Promising; limited clinical data
Degradable PEG hydrogels	Crosslinked PEG systems	Tunable	Low-MW PEG fragments	Renal clearance	Favorable when fully degradable
Non-degradable structural polymers	PMMA, PEEK, PEKK	Non-degradable	None	Fibrous capsule formation; long-term persistence	Used when permanence acceptable
Conductive/electroactive polymers	PVDF, PEDOT, PANI	Non-degradable	None	Persistent materials; encapsulation risk	Undesirable for transient regeneration
Carbon-based composites	CNTs, graphene, fullerenes, quantum dots	Non-degradable	None	Partial clearance or tissue accumulation	Safety concerns limit translation
Magneto/ultrasound-active fillers	SPIONs, BaTiO3, piezoceramics	Non-degradable	None	Slow macrophage-mediated clearance	Preclinical; no FDA approval

## Data Availability

The data that support the findings of this study are available on request from the corresponding author. The data are not publicly available due to privacy or ethical restrictions.
